# Development and characterization of triazole-based WDR5 inhibitors for the treatment of glioblastoma

**DOI:** 10.1172/jci.insight.198298

**Published:** 2026-05-05

**Authors:** Jesse A. Coker, Steven R. Martinez, Sang Hoon Han, Anthony R. Sloan, Amit Kumar Gupta, George Bukenya, Paul Polzer, James H. Ramos, Emma G. Rico, Annabella Rico, A. Abigail Lindsey, Tanvi Navadgi, Natalie Reitz, Todd Romigh, Jonathan Macdonald, Dhiraj Sonawane, Christopher M. Goins, Christopher G. Hubert, Nancy S. Wang, Feixiong Cheng, Joseph Alvarado, Samuel A. Sprowls, Justin D. Lathia, Shaun R. Stauffer

**Affiliations:** 1 Department of Molecular Medicine, Cleveland Clinic Lerner College of Medicine of Case Western Reserve University, Cleveland, Ohio, USA.; 2 Cleveland Clinic Center for Therapeutics Discovery (C3TD), Cleveland Clinic Research, Cleveland, Ohio, USA.; 3 College of Pharmacy, Korea University, Sejong, Korea.; 4 Department of Cardiovascular Medicine, Cleveland Clinic Research, Cleveland, Ohio, USA.; 5 Case Comprehensive Cancer Center, Case Western Reserve University, Cleveland, Ohio, USA.; 6 Cleveland Clinic Genome Center and; 7 Genomic Medicine Institute, Cleveland Clinic Research, Cleveland, Ohio, USA.; 8 Department of Biochemistry, Case Western Reserve University, Cleveland, Ohio, USA.; 9 PASS Division and; 10 Graudate School of Pharmaceutical Sciences, Duquesne University School of Pharmacy, Pittsburgh, Pennsylvania, USA.; 11 Rose Ella Burkhardt Brain Tumor & Neuro-Oncology Center, Cleveland Clinic Research, Cleveland, Ohio, USA.

**Keywords:** Cell biology, Neuroscience, Oncology, Brain cancer, Structural biology, Therapeutics

## Abstract

Glioblastoma (GBM) cancer stem cells (CSCs) contribute to tumor recurrence, treatment resistance, and dismal clinical outcomes. Genetic and pharmacological evidence suggests that the nuclear scaffolding protein WD-repeat containing protein 5 (WDR5) is a therapeutic vulnerability of the CSC population. However, previously reported WDR5 inhibitors display low permeability and are unable to penetrate the blood-brain barrier (BBB), limiting their utility in GBM. Herein, we report the structure-guided development of a series of triazole-based WDR5 WIN-site inhibitors designed to increase passive brain penetration. We identified triazole-based WDR5 inhibitors that are potent, passively permeable, and in some cases more brain penetrant than other scaffolds. We phenotypically assessed our WDR5 inhibitors in a panel of patient-derived CSC models and uncovered unique WDR5-regulated metabolic genes in GBM. We also evaluated their antiproliferative activity against CSCs both in vitro and in vivo. Finally, to identify potential combination opportunities, we screened a 2,100-compound chemical probe library and identified that the ATAD2 inhibitor BAY-850 synergizes with WDR5 inhibitors to enhance CSC killing. Our work diversifies the chemical matter targeting WDR5, clarifies the in vitro consequences of WIN-site inhibition in CSCs, and encourages the future development of next-generation WDR5 inhibitors with the potential to achieve in vivo efficacy in the brain.

## Abstract 2

We synthesized new WDR5 inhibitors and characterized their ability to enter the brain and treat glioblastoma.

## Introduction

Despite an aggressive treatment regimen consisting of surgical resection, radiation, and chemotherapy, the prognosis for glioblastoma (GBM), the most common primary malignant brain tumor, is dismal: median survival is 14–16 months for patients eligible for clinical trials, and 5-year overall survival is less than 3% (1–3). There remains an urgent need for next-generation targets and treatment paradigms that specifically address the underlying challenges of GBM: innate therapeutic resistance, pronounced tumor heterogeneity and cellular plasticity, and the persistence of self-renewing cancer stem cells (CSCs) that drive rapid tumor recurrence and mortality (4–10). CSCs display enhanced DNA damage response, hyperproliferative capacity, resistance to conventional chemotherapies, and adaptive enrichment in specific hypoxic, tumorigenic GBM niches (11). A growing body of evidence supports the hypothesis that specifically targeting self-renewing CSCs via novel therapeutic mechanisms could improve GBM patient outcomes (12–15).

WDR5 is a core scaffolding component of the “WRAD” complex that positions mixed linear leukemia (MLL) family histone methyltransferases for deposition of the activating H3K4Me3 epigenetic mark (16–23). We previously discovered that WDR5 is functionally important for CSC maintenance in GBM: genetic deletion or pharmacological inhibition of WDR5 reduced the viability and self-renewal capacity of CSC populations (24). These findings align with other reports demonstrating that dpy-30 histone methyltransferase complex regulatory subunit (DPY30) and Retinoblastoma-binding protein 5 (RBBP5), 2 other WRAD complex members, are genetic dependencies in CSCs (25, 26).

**Figure 1:**
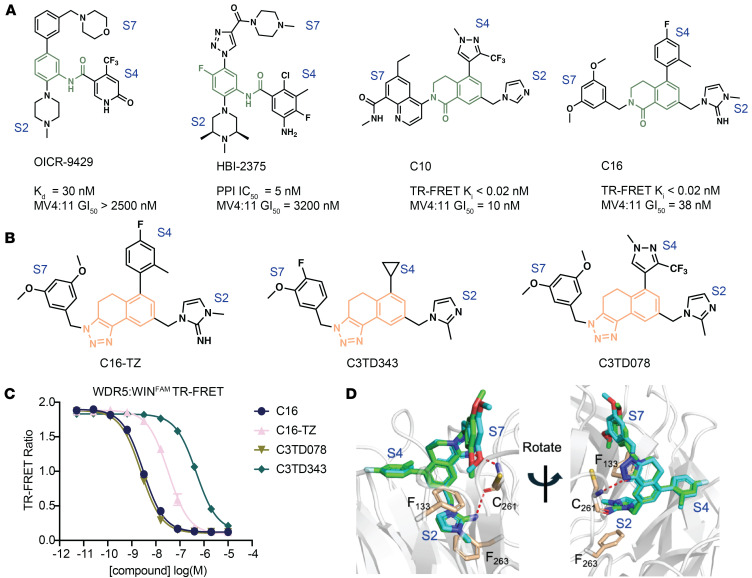
Biochemical and structural characterization of triazole-based WDR5 inhibitors. (**A**) Structures and potencies of previously reported WDR5 WIN-site inhibitors. (**B**) Structures of WIN-site inhibitors synthesized herein. (**C**) A biochemical TR-FRET assay was used to measure compound-dependent displacement of a labeled WIN-site peptide from human WDR5. Representative data from a single replicate performed in technical quadruplicate are presented as mean ± SD. (**D**) Overlay of WDR5:C16 (green; PDB: 6UCS) and WDR5:C16-TZ (cyan, PDB: 9NCW) cocrystal structures reveals a conserved binding mode at the WIN-site, including especially a bidentate H-bond to Cys261.

Independently of changes to H3K4Me3 levels, small-molecule inhibitors of WDR5’s WDR5-interacting (WIN) site disrupt the protein-protein interaction (PPI) between WDR5 and MLL1, evict WDR5 from chromatin, reduce MYC-target gene expression, downregulate a core set of 50–100 ribosomal protein genes (RPGs), and reduce bulk cellular translation capacity (27–33). WIN-site inhibitors, such as the best-in-class imidazole-based compounds C16 and C10 (Figure 1A), achieve potent and robust single-agent in vivo antiproliferative activity against MLL-rearranged (MLLr) leukemias that depend on oncogenic amplification of MLL1 (34–37). Beyond these relatively rare MLLr leukemias, WDR5 inhibition has been implicated as a promising therapeutic approach in a diverse set of hematological malignancies and solid tumors—specifically including both GBM and rhabdoid tumors in the brain (24, 32, 37–55). However, despite promising in vitro activity, there are still no reports of a potent, brain-penetrant WDR5 inhibitor, preventing in vivo validation of WDR5 inhibition as a therapeutic strategy in any neurooncological indication (56, 57).

Our goal was to generate potent, brain-penetrant WDR5 WIN-site inhibitors by reengineering the core dihydroisoquinolinone scaffold of compounds like C16. We were inspired to remove C16’s lactam, reported to be a liability for BBB penetration in the β-secretase (BACE-1) field, and replace it with a fused heterocyclic system to improve permeability. Herein, we describe a limited series of WDR5 inhibitors containing a tricyclic triazole as a core amide isostere. These triazole-based inhibitors potently inhibit WDR5, maintain a conserved binding mode in the WIN-site, exhibit passive permeability, and, in some cases, improve brain penetration. We demonstrated that these triazole analogs potently engage WDR5 in patient-derived (PD) CSC models, and we characterized their mechanism of action and potential for use in combinatorial regimens. Our efforts revealed that WIN-site inhibitors downregulate unique metabolic transcripts in CSCs and can be combined with the ATAD2 inhibitor BAY-850 to enhance CSC killing. Although we gained in vitro mechanistic insights using these tool compounds, our most advanced triazoles did not reduce tumor growth or improve overall survival in vivo, likely due to insufficient potency, brain exposure, and/or metabolic stability. Our work reveals GBM-specific effects of WIN-site inhibition, diversifies the relatively limited chemical matter against WDR5, and provides proof-of-concept that WDR5 inhibitors can indeed be redesigned for improved BBB penetration.

## Results

### Design and characterization of tricyclic triazole-based WIN-site inhibitors.

Previously reported WDR5 WIN-site inhibitors contain a tri-substituted amide core with an Arg-mimicking “S2” group, which penetrates to the bottom of the WD40-domain central cavity, and 2 additional “S4” and “S7” substituents that make interactions with pockets along the surface of WDR5 (Figure 1A). The only brain-penetrant WDR5 inhibitor reported to date is HBI-2375 (56), a methylpiperazine-S2-containing WIN-site binder disclosed in a patent owned by Huyabio (Figure 1A) (58). However, HBI-2375 is not potent, displaying GI_50_ > 3 μM against the sensitive MLLr cell line MV4:11. Therefore, we initiated our medicinal chemistry campaign from a more potent starting point, namely C16 and C10 (Figure 1A), which incorporate an imidazole-based S2 discovered with fragment-based methods by Fesik and colleagues (31, 34, 35, 37, 59). C16 and C10 were reported to have a direct WDR5 K_i_ < 20 pM and an MV4:11 GI_50_ = 38 nM and 10 nM, respectively (Figure 1A) (37). However, our in vitro profiling of C16 indicated poor passive permeability (Table 1), and exploratory in vivo rodent experiments confirmed that C16 could not penetrate the BBB (brain/plasma ratio [P:B] < 0.05; see Supplemental Figure 1; supplemental material available online with this article; https://doi.org/10.1172/jci.insight.198298DS1).

To reengineer C16 into a brain-penetrant compound, we identified the core dihydroisoquinolinone as a potential moiety restricting passive permeability. Compounds like C16 incorporate a 1,3,5-trisubstited isophthalate scaffold, which was heavily investigated in the BACE-1 field for Alzheimer’s disease. Despite significant efforts, only a few isophthalates were shown to have any measurable brain penetration (60–62). Based on reports that an oxadiazole bioisostere could improve the BBB permeability of such BACE-1 scaffolds, we were inspired to replace the lactam of C16 with a fused heterocycle (62–64). Our approach centered on constraining the *N*-benzylic lactam within a fused ring system, extending a flexible vector toward the S7 pocket, and maintaining a key backbone hydrogen bond between the core and Cys261. We identified a tricyclic triazole as a promising scaffold (Figure 1B) that removed the central amide while retaining excellent on-target potency (Figure 1C and Table 1). Consistent with our design hypothesis, the tricyclic triazoles showed modestly improved predicted permeability relative to C16 as scored by both the central nervous system molecular properties optimization (MPO) and BBB algorithms (Table 1) (65–67).

To understand the relative properties of triazole- versus dihydroisoquinolinone-based WIN-site inhibitors, we synthesized the matched pair of C16, C16-TZ (Figure 1B). We characterized this pair in a biochemical time-resolved fluorescence resonance energy transfer (TR-FRET) assay, which measures the displacement of a labeled WIN-peptide from recombinant human WDR5 (Figure 1C and Table 1). With an imidazole-imine S2, the triazole C16-TZ was sub-nanomolar (K_i_ = 0.37 nM); however, this represented a 12-fold potency loss versus C16, which demonstrated picomolar activity (K_i_ = 0.03 nM) consistent with previously reported values (37). Guided by prior S2 SAR from Fesik and colleagues (37), we synthesized analogs with a 2-methyl imidazole S2 and a 3-methoxy phenyl S7 (Figure 1B) that were predicted to have improved permeability. We prepared analogs with 2 distinct S4 groups: a low molecular weight cyclopropyl (C3TD343, K_i_ = 5.9 nM) and an elaborated trifluoromethyl pyrazole (C3TD078, K_i_ = 0.030 nM). As expected, the congener C3TD078 was found to be more potent, with an equivalent K_i_ to the comparator C16 (Figure 1C and Table 1).

To confirm the structural basis of the triazole isostere, we solved a high-resolution 1.7 Å crystal structure of WDR5 bound to C16-TZ and validated a conserved binding pose matching C16 at the WIN-site (PDB: 9NCW, Supplemental Figure 2). C16 forms a bidentate hydrogen bond with the backbone of Cys261, while the imidazole-imine S2 group π-π stacks between the aromatic sidechains of Phe133 and Phe263. Figure 1D shows the overlay of C16 (PDB:6UCS) with our crystal structure of C16-TZ, wherein the tricyclic triazole core of C16-TZ maintains C16’s bidentate hydrogen bond with Cys261 as well as the relative orientations of the S2/S4/S7 substituents. We further validated this binding pose with 2 other high-resolution cocrystal structures of triazole-based WIN-site inhibitors containing methyl-imidazole S2s (Protein Data Bank: 9NCT, 9NCV; Supplemental Figure 3).

### DMPK profiling of triazole-based WDR5 inhibitors.

The calculated molecular properties and DMPK profile for triazole-based inhibitors C16-TZ, C3TD343, and C3TD078 were directly compared with the matched dihydroisoquinoline C16 (Table 1). Compounds with an imidazole-imine S2, such as C16 and C16-TZ, displayed low permeability in MDCK cells, high plasma protein binding (>99%), and moderate to high clearance in rat and human microsomes. The triazole analogs C3TD343 and C3TD078 were found to have favorable permeability (Madin-Darby canine kidney cells [MDCK] *P*_app_ > 2) and measurable free fraction of ~2%. Triazole-containing inhibitors consistently exhibited poor metabolic stability in vitro (microsomal intrinsic clearance [CL_int_] > 300 mL/min/kg).

**Figure 2:**
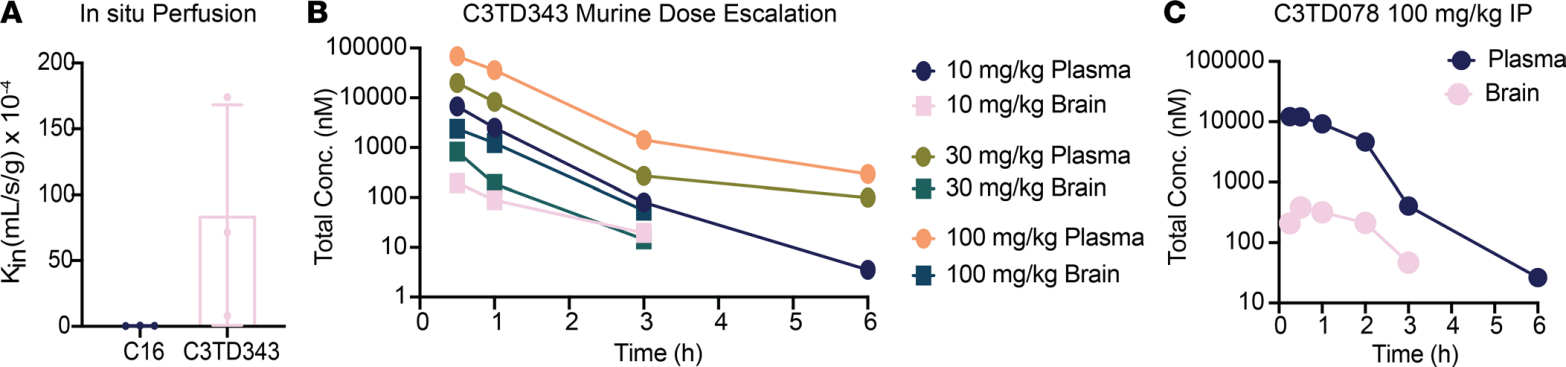
In vivo murine PK. (**A**) Unidirectional brain uptake constants for C16 and C3TD343 in murine brain perfusion experiments (*n* = 3). (**B**) C3TD343 total plasma (circles) and brain (squares) exposure after IP dose escalation from 10–100 mg/kg in mice. (**C**) Total plasma and brain exposure of C3TD078 following a single 100 mg/kg i.p. dose in mice.

Next, we moved forward with in vivo testing to assess the BBB permeability of triazole-based WDR5 inhibitors that displayed enhanced MDCK permeability. We first characterized the passive permeability of WDR5 inhibitors in 2-minute in situ brain perfusion experiments, in which compounds were directly perfused into murine hearts following ligation of the descending aorta and severing of the right ventricle. After measuring the total amount of compound entering the brain at short time points, the instantaneous rate of BBB passage was quantified as a unidirectional brain uptake constant (K_in_). C16 did not rapidly enter the brain in this perfusion setup, with a K_in_ < 5 × 10^–4^ mL/s/g (Figure 2A). Encouragingly, however, the triazole C3TD343 was readily brain penetrant and displayed an order of magnitude higher rate of permeation than C16 (K_in_ > 50 × 10^–4^ mL/s/g; Figure 2A). Overall, the permeability we observed experimentally matched the predictions from BBB and MPO scores: both algorithms scored the permeable compound C3TD343 (3.8/3.4) higher than the impermeable analogs C3TD078 (3.0/2.8) and C16 (2.9/2.5; Table 1).

We proceeded with a dose-escalation experiment of C3TD343 in mice, which revealed a dose-proportional increase in brain exposure after i.p. administration using 10, 30, and 100 mg/kg (Figure 2B). At 100 mg/kg, C3TD343 achieved a total brain C_max_ = 2.4 μM and AUC = 2.9 μM*hrs, yielding a moderate P:B = 0.25. In agreement with the in vitro CL_int_ values (Table 1), C3TD343 was rapidly cleared from circulation, leading to less than 4 hours of brain exposure. The extremely fast intake kinetics but moderate equilibrium P:B ratio for C3TD343 was indicative of active export, and indeed we found that C3TD343 was strongly effluxed by P-glycoprotein (Pgp aka MDR1) in MDR1-MDCK permeability studies (Supplemental Figure 4). In summary, some triazole-based WDR5 inhibitors like C3TD343 are quite passively permeable, but their total brain exposure was limited by Pgp-mediated export and rapid hepatic clearance.

C3TD343 contains a truncated cyclopropyl S4 substituent, which confers better brain penetration but compromises potency. Compounds with an elaborated S4, such as the CF_3_ pyrazole in C3TD078, were up to 200-fold more potent than C3TD343 (K_i_ = 0.03 nM vs 5.9 nM; Table 1). Unfortunately, the addition of polar surface area and hydrogen-bond donors within the S4 region compromised BBB penetration: when dosed in mice at 100 mg/kg by IP, C3TD078 was far less brain penetrant than C3TD343 (brain C_max_ = 406 nM, P:B < 0.05; Figure 2C). In conclusion, while we succeeded in synthesizing triazole-based WDR5 inhibitors with K_i_ < 40 pM, such potent compounds did not match the brain penetration of the moderately potent compound C3TD343.

### Transcriptional profiling of WDR5 inhibitors in GBM.

**Figure 3:**
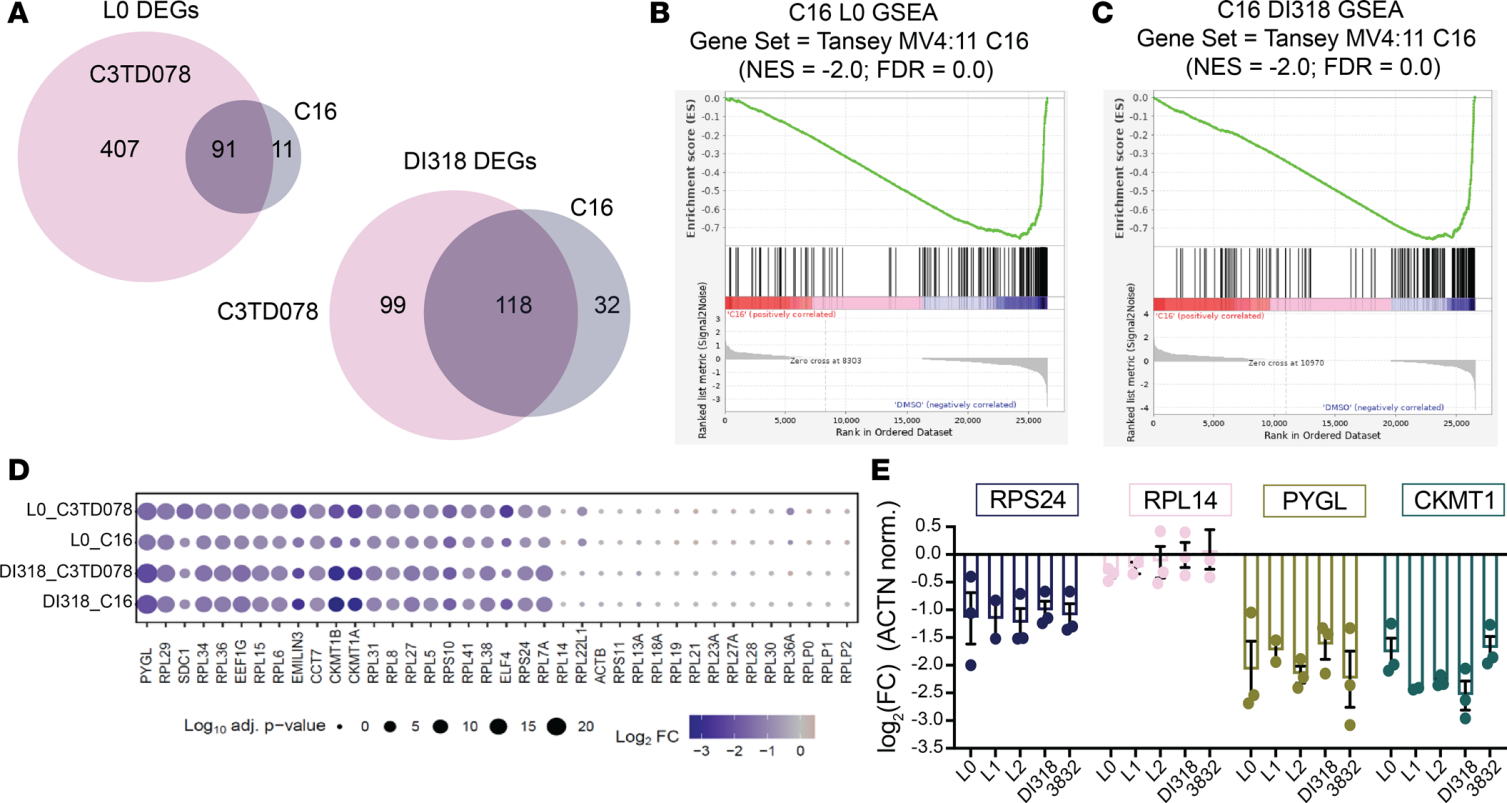
Transcriptional profiling of CSCs treated for 72 hours with 200 nM of different WIN-site inhibitors. (**A**) Overlap of differentially downregulated genes (log_2_FC < –0.5, *P* < 0.01) by C16 and C3TD078 treatment in L0 and DI318 CSCs as measured by bulk RNA-seq. (**B** and **C**) GSEA against the set of genes downregulated by C16 in MV4:11 leukemia cells, as reported by Howard et al. (33), for C16 in L0 CSCs and in DI318 CSCs. (**D**) Dot plot highlighting a representative set of *n* = 20 protein-coding genes observed to be downregulated (left) or unaffected (right) by both C16 and C3TD078 in both L0 and DI318 CSCs. (**E**) qPCR validation for the WIN-site dependent genes *RPS24*, *PYGL*, and *CKMT1* as well as the WIN-site independent gene *RPL14*. Expression (log_2_FC) relative to DMSO is reported as mean ± SEM for *n* = 2 (L1) or *n* = 3 (L0, L2, DI318, 3832) biological replicates.

Aligned with previous reports (33, 38), we treated L0 and DI318 PD CSCs for 72 hours with a noncytotoxic dose (200 nM) of C3TD078 or C16 and then profiled the transcriptional responses by bulk RNA-seq (Supplemental Figure 5). We observed a strong overlap between triazole- and dihydroisoquinolinone-based WDR5 inhibitors: in L0 cells, 91/102 (89%) differentially expressed genes (DEGs; log_2_FC < –0.5 and *P* < 0.01) from the C16 treatment were also downregulated by C3TD078 (Figure 3A); in DI318 cells, 118/150 (79%) of the C16 DEGs were also downregulated by C3TD078 (Figure 3A). Gene set enrichment analysis (GSEA) against a set of MV4:11 C16-regulated genes revealed strong downregulation of the gene set by C16 in both L0 (NES = –2.0, FDR = 0.0, Figure 3B) and DI318 CSCs (NES = –2.5, FDR = 0.0; Figure 3C), confirming that C16 modulates a consistent set of genes between CSCs and MV4:11 leukemia cells (33, 68). This overlap was driven primarily by known WDR5-regulated RPGs, which were downregulated at least 2-fold by both WIN-site inhibitors in both CSC models (Figure 3D). In total, we observed *n* = 43 protein-coding genes that were downregulated by both C16 and C3TD078 in both CSC lines and can be considered the “core” set of WIN-site dependent CSC transcripts (Supplemental Figure 6). The most significantly enriched GO term among the DEGs was “cytoplasmic translation” (GO:0002181; *P* < 2 × 10^–16^) and Reactome pathway analysis using the “core” set of 43 genes revealed strong enrichment of “translation,” “rRNA processing,” and “peptide chain elongation” (*P* < 3.3 × 10^–16^; Supplemental Figure 7). Therefore, even in the distinctive CSC cellular context, our RNA-seq data are parsimonious with the hypothesis that WIN-site inhibitors act primarily as translational stressors that deplete the ribosomal inventory (29–32, 38). Unlike in MLLr leukemias, we did not observe consistent downregulation of *RPL22L1* (Figure 3D), nor did we see consistent activation of p53 signaling (only C16, but not C3TD078, weakly enriched GO terms related to p53; Supplemental Figure 5) (33).

In addition to changes in conserved WDR5-regulated RPGs, we identified *n* = 8 genes that were downregulated in CSCs but not in MV4:11 leukemia cells. These included 2 additional RPGs (*EEF1G*, *RPS18*), a transcription factor (*ELF4*), and 2 extracellular matrix proteins (*SDC1*, *EMILIN3*). Most intriguingly, we also observed a GBM-specific metabolic phenotype of WIN-site inhibitors, with *PYGL* (encoding the liver isoform of glycogen phosphorylase) and *CKMT1A/1B* (both encoding the mitochondrial creatine kinase CKMT1) being strongly and consistently downregulated (Figure 3D). CKMT1 synthesizes phosphocreatine, a metabolite that is upregulated in CSCs and that directly drives oncogenic epigenetic reprograming (69). *PYGL* was the single most significantly downregulated gene across both compounds in both CSC lines despite being unaffected by C16 in MV4:11 leukemia cells (Supplemental Figure 5) (33). The “liver” PYGL isoform, which performs the rate-limiting step of the cytosolic glycogen-shunt that mobilizes glucose to fuel cancer cell growth (70, 71), is known to be overexpressed in GBM, and genetic deletion of PYGL has been shown to enhance GBM radiosensitivity (72). We did observe a modest enhancement in the antiproliferative activity of C3TD078 following 2 Gy irradiation, but only in a single CSC line (DI318; Supplemental Figure 8).

We extended these RNA-seq observations by qPCR validation in 5 different PD CSC models (Figure 3E). The WDR5-bound, WIN-site responsive RPG, *RPS24*, was downregulated ~2-fold by C3TD078 treatment in all 5 models, while the WDR5-independent RPG *RPL14* was unaffected, as expected and reported by others (31). In agreement with our RNA-seq data, both *PYGL* and *CKMT1* were downregulated by ~4-fold in all 5 CSC models (Figure 3E). We also validated that the “liver” *PYGL* isoform is expressed at > 1,000 fold higher levels than the “brain” *PYGB* isoform in CSCs, and we found that WIN-site inhibitors had little to no effect on the already low expression of *PYGB* (Supplemental Figure 9). Taken together, our transcriptional profiling revealed both ubiquitous RPGs and context-specific metabolic transcripts (*PYGL*/*CKMT1*) that were downregulated by WIN-site inhibition in CSCs.

### Target engagement of WDR5 inhibitors in CSCs.

**Figure 4:**
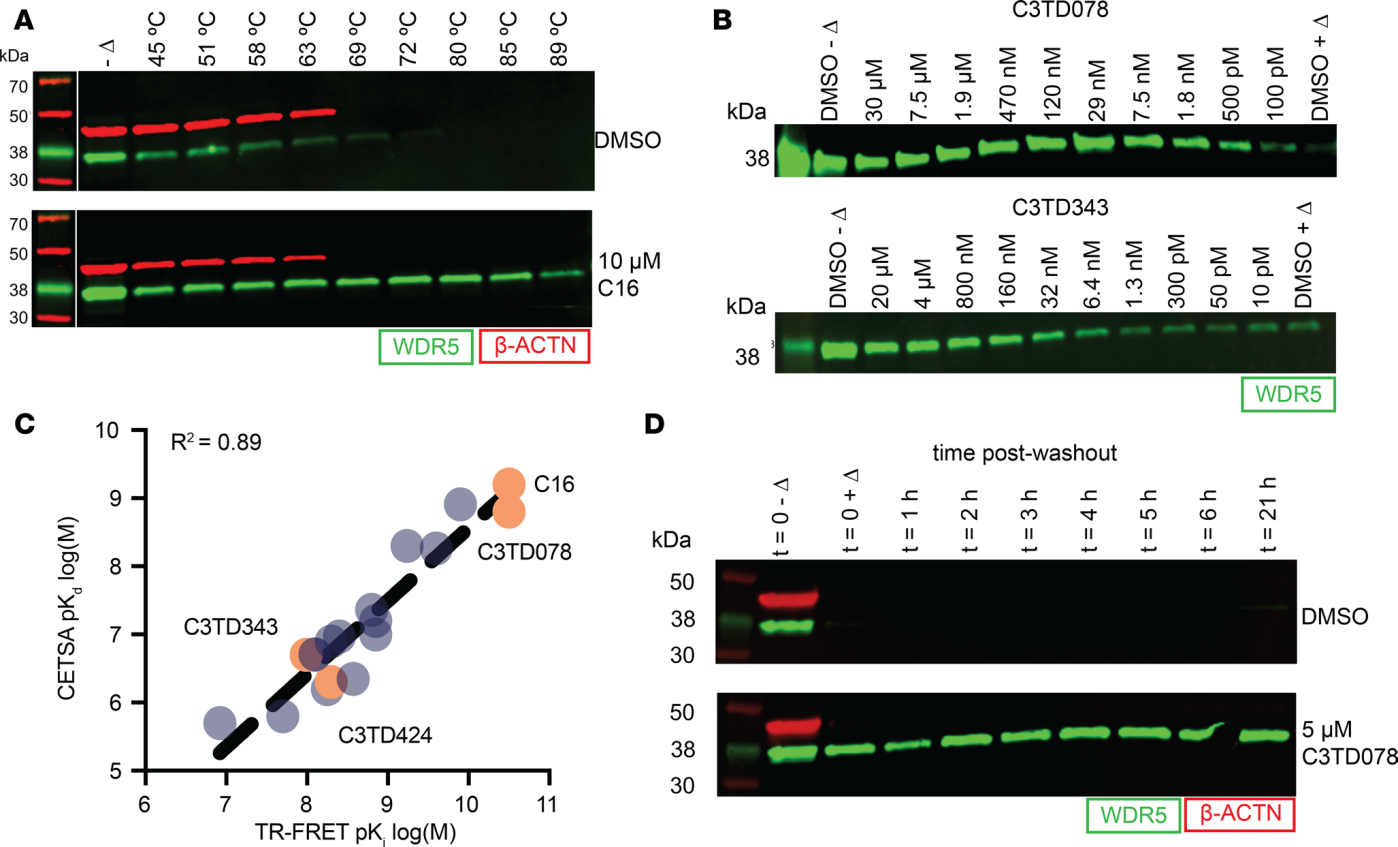
Direct WDR5 target engagement assessed by CETSA. (**A**) Melting curve for WDR5 (green) and ACTB (red) determined by CETSA-WB following a 2-hour treatment of L0 CSCs with either DMSO (top) or 10 μM C16 (bottom). (**B**) Isothermal CETSA-WB (70°C) for L0 CSCs treated for 2 hours with varying doses of the potent binder C3TD078 (top) or the weaker binder C3TD343 (bottom). (**C**) Correlation between biochemical potency (TR-FRET K_i_) and cellular potency (L0 CETSA K_d_) as determined for *n* = 17 different WDR5 inhibitors. (**D**) Washout experiment following the pretreatment of L0 CSCs for 2 hours with either DMSO (top) or 5 μM C3TD078 (bottom). Samples were prepared for isothermal CETSA-WB at the indicated timepoints post-washout.

To confirm intracellular target engagement of our WDR5 inhibitors in CSCs, we first used a cellular thermal shift (CETSA) assay in intact L0 CSCs. In total, 10 μM of C16 induced a ΔT_m_ = +35°C in L0 cells but did not change the T_m_ of β-actin (Figure 4A). For intracellular K_d_ determinations, we performed compound titrations (2-hour pretreatment) with an isothermal melt at 70°C followed by centrifugation at 14,000 × *g* to separate ligand-bound from apo WDR5. Representative data for a potent compound (C3TD078, K_d_ = 2 nM) and a weak compound (C3TD343, K_d_ = 200 nM) are provided in Figure 4B. Across all tested triazoles (*n* = 17), we observed a linear correlation (*R*^2^ = 0.89) between the biochemical K_i_ as determined by TR-FRET and the intracellular K_d_ as determined by CETSA (Figure 4C). We also deployed the CETSA in washout experiments to establish the profoundly long off rate of C3TD078, which remained bound to WDR5 for at least 21 hours following compound removal (Figure 4D). C16 also remained bound to WDR5 for > 20 hours following washout (Supplemental Figure 10), suggesting that, across chemotypes, potent WDR5 inhibitors display a slow intracellular off rate.

**Figure 5:**
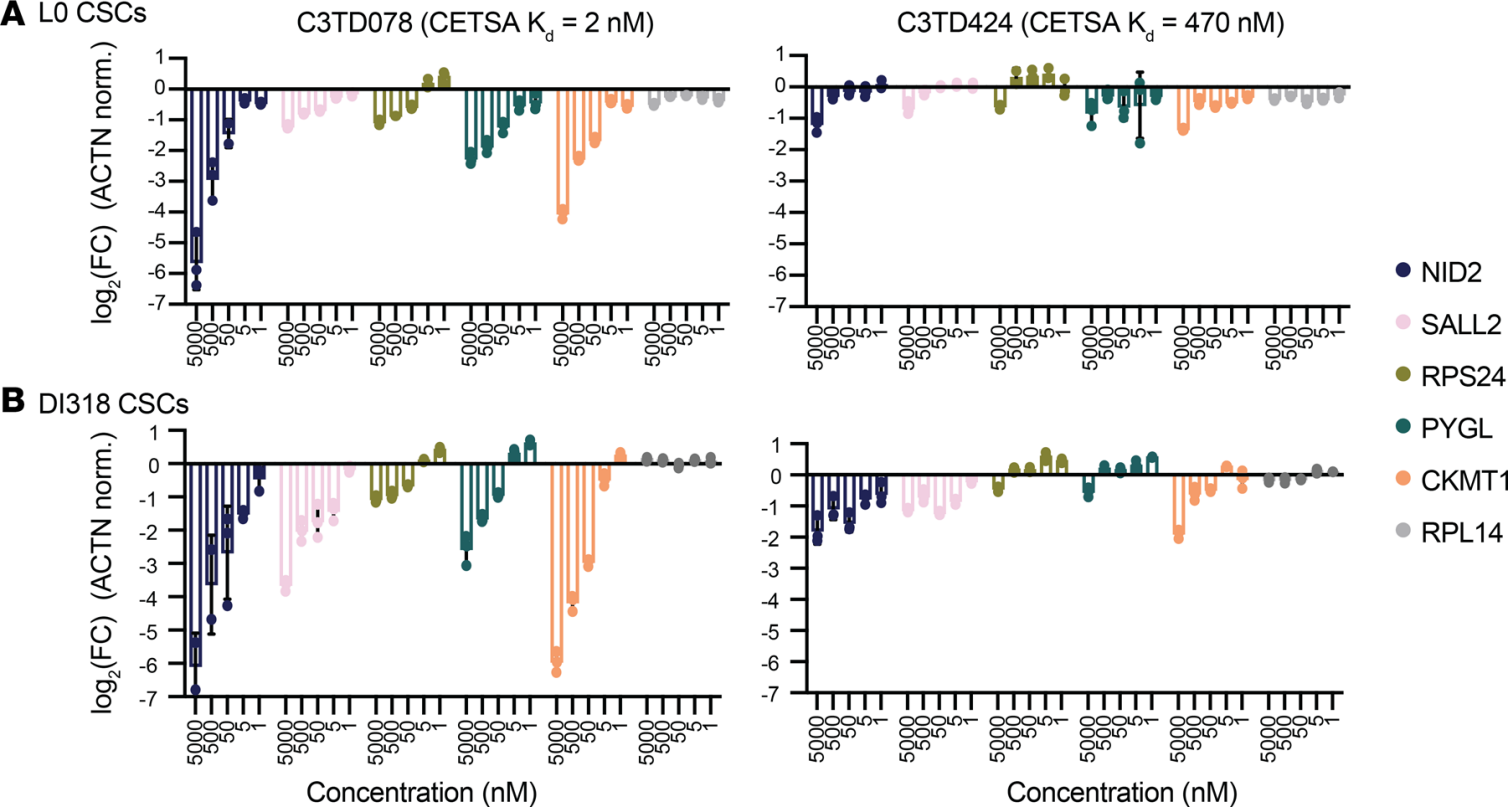
Dose responsive transcriptional inhibition with C3TD078 or C3TD424. (**A** and **B**) qPCR analysis for the indicated genes after treating L0 (**A**) or DI318 (**B**) CSCs with the indicated doses of the potent inhibitor C3TD078 (left) or the weak inhibitor C3TD424 (right) for 72 hours. Data are presented as mean ± SD from a single biological replicate completed in technical triplicate.

To further validate the on-target nature of the transcriptional effects observed in Figure 3, we performed head-to-head dose-response experiments by qPCR using both a potent (C3TD078) and weak WDR5 inhibitor (C3TD424, CETSA K_d_ = 500 nM; Supplemental Figure 11). In addition to the RPGs and metabolic transcripts identified by RNA-seq in Figure 3, we also quantified the expression of *SALL2* and *NID2*, which were identified as WDR5-bound genes in CSCs by CHIP-seq in our previous study (24). In both L0 and DI318 cells, C3TD078 achieved potent, dose-dependent inhibition of *RPS24*, *PYGL*, *CKMT1*, *SALL2*, and *NID2*, while the less potent C3TD424 showed only modest inhibition of these genes at only the highest doses (Figure 5). As expected, neither compound affected the expression of the WDR5-independent gene *RPL14*. Across both cell lines and all 5 genes, the average IC_50_ = 80 nM for C3TD078-induced transcriptional downregulation, which closely matches reported IC_50_ values for C16’s transcriptional activity in MLLr leukemia cells (38). Taken together, these qPCR data align with the CETSA and support potent on-target inhibition of WDR5 by triazole-based WIN-site inhibitors in CSCs.

### Antiproliferative activity of triazole-based WDR5 inhibitors against CSCs.

In MLLr-leukemia cell lines like MV4:11, potent WIN-site inhibitors like C16 achieve low double-digit nM antiproliferative activity, while in insensitive cell lines, like A549, the same WIN-site inhibitors display GI_50_ > 2 μM (35, 59). In a 7-day assay using 5 different CSC models, neither C16 nor C3TD078 achieved remarkable antiproliferative activity, with GI_50_ values between 1 and 5 μM (Table 2). There was a trend toward more potent GI_50_ values with C3TD078/C16 relative to the weaker compound C3TD343, suggesting a modicum of on-target, WDR5-mediated antiproliferative activity across this CSC panel. However, we also observed that both C16 and C3TD078, likely due to their protonatable S2 warheads, induced phospholipidosis (PLD) and disrupted cellular membrane architecture at doses above 1 μM (Supplemental Figure 12). Therefore, we could not rule out the possibility of off-target PLD dominating the observed antiproliferative phenotype (73–75).

**Figure 6:**
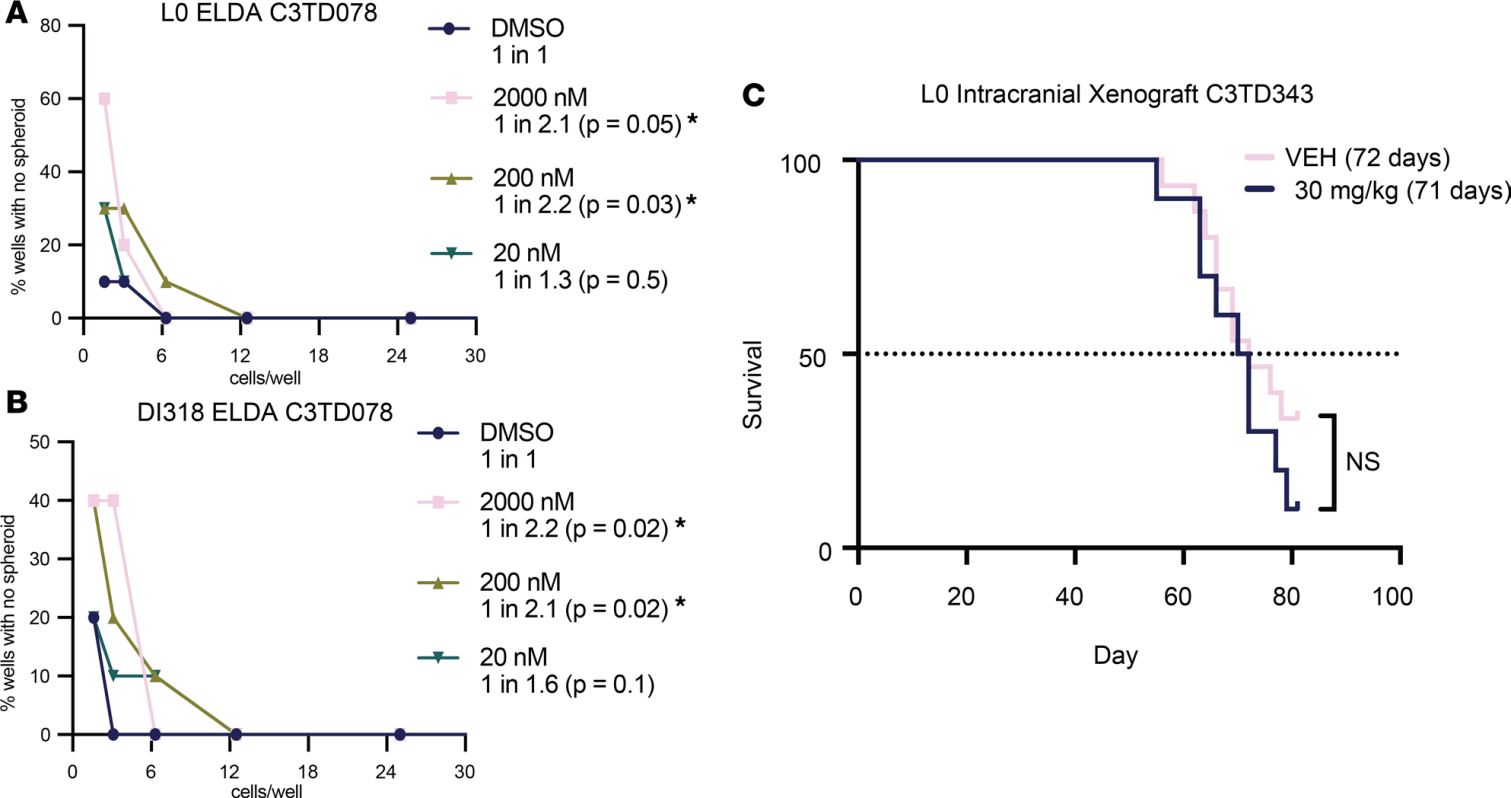
Effects of long-term WDR5 inhibition on CSC growth. (**A** and **B**) ELDA after constant treatment of L0 (**A**) or DI318 (**B**) CSCs for 14 days with the indicated doses of C3TD078. Representative data from one of 2 biological replicates are shown; each point represents the fraction of nonresponding wells out of 10 technical replicates at each cell number. Stem cell frequency and *P* value relative to DMSO were calculated as previously reported (98). *P < 0.05. (**C**) Kaplan-Meier survival curve for mice implanted with intracranial L0 PDX tumors and treated daily with vehicle or 30 mg/kg once a day i.p. C3TD343.

Next, we examined the ability of C3TD078 to reduce the stem-cell frequency of CSCs with a traditional extreme limiting dilution assay (ELDA). We observed that C3TD078 dose-dependently and significantly decreased the stem-cell frequency of L0 (Figure 6A) and DI318 (Figure 6B) CSCs, but only modestly — 200 nM of C3TD078 resulted in a halving of the number of stem cells. These effects on self-renewal were much smaller than previously reported with 5 μM of C16, perhaps because of WDR5-independent cytotoxicity at this high dose (24).

Finally, we characterized our most brain-penetrant but modestly potent compound C3TD343 in an intracranial xenograft. We treated mice implanted with intracranial L0 PDX tumors for up to 71 days with 30 mg/kg once a day i.p. C3TD343, which was well tolerated. However, we observed no effect on survival (Figure 6C).

### Synergy between WDR5 and ATAD2 inhibitors against CSCs.

**Figure 7:**
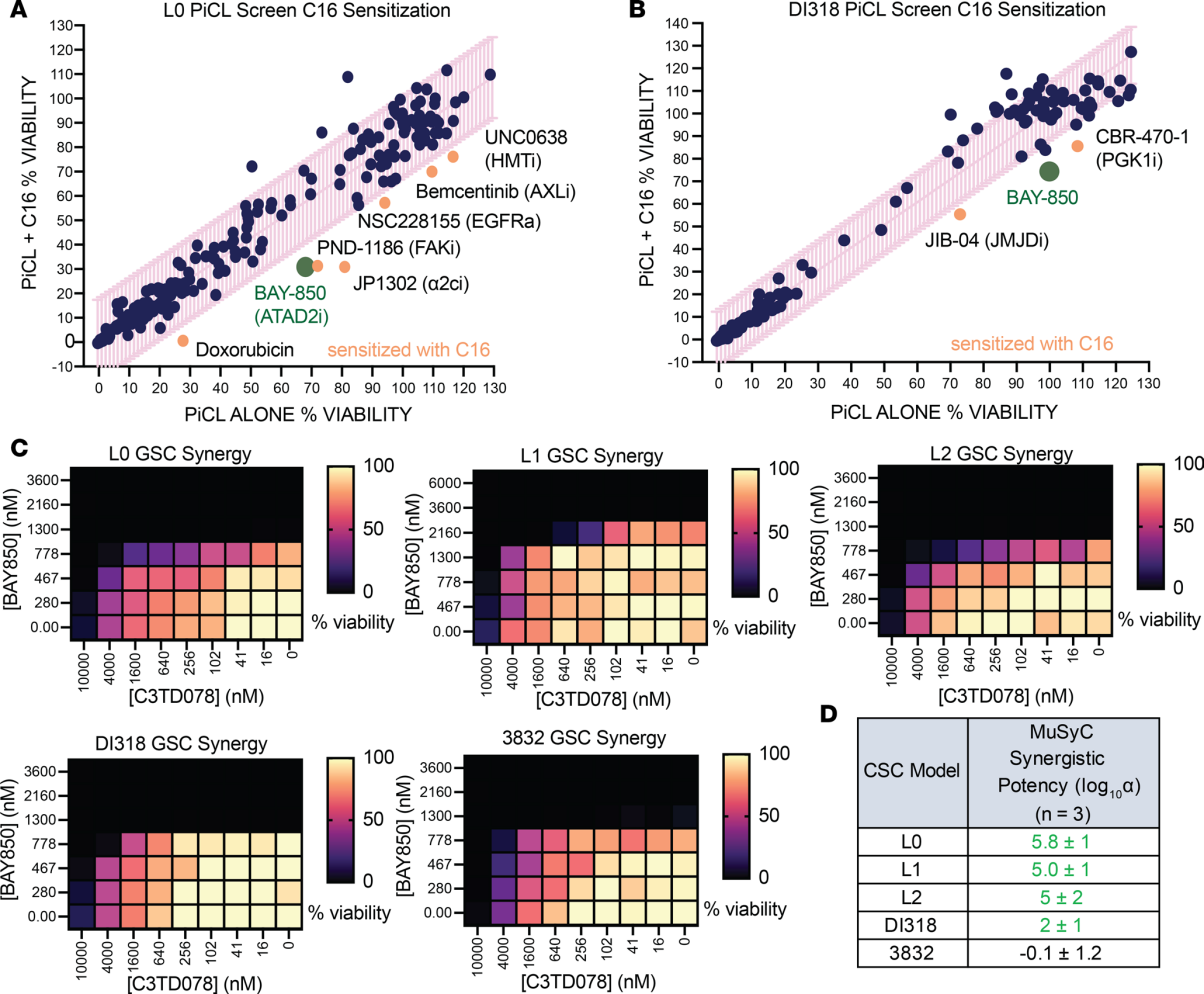
High-throughput screening reveals synergy between ATAD2 and WDR5 inhibition. (**A**) Synergy screen with the Pinpoint Chemical Probe Library (PiCL; 1 μM) by CTG viability after 7-day treatment in the presence (*y* axis) or absence (*x* axis) of 100 nM C16. The 95% CI of the linear regression is displayed in pink. PiCL library members that reduced the viability of CSCs more in the presence of C16 are toward the southeast of the plot and are labeled. (**B**) Same as **A**, except in DI318 CSCs. (**C**) Seven-day CTG viability cross-titrations with C3TD078 and the ATAD2 inhibitor BAY-850 in 5 different CSC models. Data are representative from 1 of 3 biological replicates. (**D**) Overall quantification of the synergistic potency, as assessed by the MuSyC algorithm across all 3 biological replicates, between BAY-850 and C3TD078 in CSCs.

Since WIN-site inhibitors did not robustly kill CSCs, we decided to pursue a combination approach, which is precedented: the BCL-2 inhibitor Venetoclax synergizes with WDR5 inhibitors to kill leukemia cells (36), while the p53 activator Nutlin-3A enhances the efficacy of WDR5 inhibitors against rhabdoid tumors (38). However, to our knowledge, there have been no reports of an unbiased synergy screen to identify combination opportunities from a large library. We were further encouraged to consider broad screening since we observed no synergistic efficacy between Venetoclax, the p53 activator RG7112, or other rational combinations with WDR5 inhibitors in our CSC models (Supplemental Figure 13). Therefore, we screened the Cleveland Clinic’s 2,100 compound Pinpoint Chemical Probe Library (PiCL) (76), which contains FDA-approved drugs and publicly available chemical probes, for compounds that killed CSCs more effectively in the presence of the WDR5 inhibitor C16. We first screened the entire library (at 1 μM) in the presence of a noncytotoxic dose (100 nM) of C16 using a 7-day CTG viability assay in both L0 and DI318 CSCs. Any treatment achieving a > 80% reduction in cell viability in either cell line was then counter screened in the presence and absence of C16 to identify synergistic combinations. We identified 7 PiCL compounds that killed L0 cells better in the presence C16 (Figure 7A); in DI318 cells, we identified 3 combination opportunities (Figure 7B). There was a single hit shared between both cell lines: BAY-850 (77), an inhibitor of the bromodomain-containing, epigenetic regulator ATPase family AAA domain-containing protein 2 (ATAD2) (78).

After validating the expression of ATAD2 in our CSC models by Western blot (Supplemental Figure 14), we proceeded to validate the synergy suggested by the PiCL screen in 5 different CSC models by performing cross titrations with BAY-850 and C3TD078 (Figure 7C). We analyzed the dose-response matrices with the MuSyC algorithm, which can disentangle synergistic efficacy (i.e., combinatorial increases in the maximum cytotoxic effect) from synergistic potency (i.e., a left-shift in the combinatorial IC_50_, denoted α) (79). We observed a relatively steep dose-response curve with BAY-850 alone, perhaps due to its mechanism of action as an ATAD2 dimer inducer (77). Nevertheless, we did observe clear synergistic potency increases, with BAY-850 left-shifting the GI_50_ of C3TD078 (α > 1) in 4 of 5 CSC models (Figure 7D).

These data resonate with a recent report demonstrating that ATAD2 is a downstream effector of WDR5 that plays a role in the growth of acute lymphocytic leukemia cells (80). We did not observe synergy with the ATAD2 bromodomain competitive inhibitor GSK8814 (81), suggesting that the binding of ATAD2 at H4Kac is dispensable for the observed synergy (Supplemental Figure 15). We observed that BAY-850 did not affect the expression of WDR5-target genes *PYGL* and *RPS24* in L0 and L2 CSCs; in combination with C3TD078, it also did not increase the downregulation of these genes relative to C3TD078 alone (Supplemental Figure 16). Therefore, the synergistic cytotoxicity of BAY-850 appears to be independent of the expression of WDR5 target genes. Taken together, and limited to only in vitro observations, these data implicate ATAD2 dimerization as a promising mechanism for combination with WDR5 WIN-site inhibition.

## Discussion

Herein, we disclose a series of triazole-based WDR5 inhibitors designed to be passively brain penetrant for the treatment of GBM. Mechanistic studies with our triazoles confirmed potent intracellular WDR5 target engagement, while bulk RNA-seq in WDR5-inhibitor–treated CSCs revealed both ubiquitous RPGs and a set of CSC-specific transcripts (*PYGL*, *CKMT1*, *SALL2*) that were downregulated. These functional data improve upon our previous work, which reported the in vitro activity of C16 in CSCs at doses ≥ 3 μM (24), through the consistent use of lower doses of C16/C3TD078 that do not induce off-target PLD. On exemplar triazole, C3TD343, showed robust passive BBB permeability, but it was not efficacious in an intracranial xenograft due to a combination of low potency, rapid hepatic clearance, and Pgp-mediated export. We also characterized the potent triazole C3TD078 in proliferation assays against 5 GBM CSC models, but we consistently observed unremarkable antiproliferative activity. Ultimately, we never identified a triazole with both picomolar WDR5 affinity (like C3TD078) and high brain exposure (like C3TD343).

We did uncover an opportunity to potentiate the activity of WDR5 inhibitors via cotreatment with the ATAD2 inhibitor BAY-850, which we identified as a synergistic combination from a 2,100 compound chemogenomic screen. Unfortunately, BAY-850 does not display suitable DMPK properties for in vivo studies (77), precluding in vivo validation of the synergy and reducing the translational potential of a combination regimen. This is an important limitation of our study, which could be addressed in the future following the development of more advanced ATAD2 dimerizers that display suitable plasma stability and brain penetration.

Overall, our work emphasizes a considerable challenge for the field: WDR5 inhibitors must have exquisitely potent direct affinity to have any functional effects in cells (56). For example, C3TD078**^’^**s potency right-shifts from biochemical TR-FRET K_i_ = 30 pM, to cellular CETSA K_d_ = 2 nM, to functional qPCR IC_50_ = 80 nM. This represents, in total, a 2,700-fold right-shift in C3TD078’s potency from direct WDR5 binding to transcriptional downregulation. Therefore, maintaining both pM direct affinity (with molecules consistently displaying molecular weight > 500 Da) and brain penetration is a difficult hurdle, especially with the requirement for a weakly basic S2 Arg-mimetic. The S4-truncated C3TD343 does indeed cross the BBB, but like the other reportedly brain penetrant analog HBI-2375, it is too weak (K_i_ = 5 nM) to have pronounced functional effects in cells; a similar 2,700-fold shift would imply an unfeasible transcriptional IC_50_ > 13 μM, well above even the total brain exposure achievable with C3TD343 (C_max_ = 2.4 μM at 100 mg/kg).

This challenge is compounded by the modest free fraction of all WDR5 inhibitors used herein (~ 1%, Table 1), which further limits bioactive unbound drug levels in vivo. In fact, we did attempt a shorter-term flank DI318 PDX model with our most potent, slow off-rate, and peripherally bioavailable triazole C3TD078. At 30 mg/kg once a day i.p. over 7 days, C3TD078 did not reduce tumor growth or affect stem cell frequency, consistent with our in vitro data (Supplemental Figure 17, A and B). However, inconsistent with our in vitro data, we observed that C3TD078 did not downregulate WDR5-target genes in treated tumors (Supplemental Figure 17C)_._ This can be rationalized by the observation that the relevant free plasma concentration of C3TD078 exceeded the CETSA K_d_ required for binding, but not the qPCR IC_50_ required for inhibition of WDR5 target genes (Supplemental Figure 17D).

Taken together, then, it is difficult to establish with certainty whether the lack of in vivo efficacy was due limitations of the triazoles themselves or a broader lack of efficacy of WIN-site inhibition in GBM. The modest GI_50_ values in 7-day in vitro proliferation assays with C16 and C3TD078 support the hypothesis that single-agent WDR5 inhibition is not a transformative paradigm in GBM. However, it is well established that the biology of GBM is distinctive in the brain, likely due to specific microenvironmental cues that can only be recapitulated in the correct tissue. KO of DPY30, an essential member of the WRAD complex alongside WDR5, is dispensable for GBM growth in vitro but essential for in vivo intracranial GBM growth and self-renewal (25). Many other epigenetic proteins, including DOT1L, SETD2, BRD4, and JMJD6 only score as essential genes in genetic dropout screens conducted intracranially, while they are wholly dispensable for CSC growth in tissue culture (82). Therefore, because of the complex local biology and epigenetics of GBM in the brain, it remains an open question if potent WIN-site inhibitors are antiproliferative against GBM models in vivo.

We are hopeful that our report will catalyze future medicinal chemistry efforts to enable the testing of WDR5 inhibitors, perhaps alongside improved ATAD2 dimerizers, in a biologically relevant in vivo setting. Our experience suggests that more compact, lower–molecular weight WDR5 inhibitors distinct from the reported chemical space will be required. We also recognize the potential for unique formulations and routes of administration (such as lipid nanoparticle encapsulation or transient ultrasound-mediated BBB opening) that could overcome the need for a passively penetrant compound. Finally, given that WDR5 is a genetic dependency specifically in the stem-like outer rim of GBM organoids and tumors (24), it merits further evaluation of the ability of WIN-site inhibitors to reduce GBM recurrence following surgical resection. Overall, our work diversifies the known chemical matter against WDR5, clarifies the in vitro phenotype of WIN-site inhibition in CSCs, and sets the foundation for future development of brain-active WDR5 inhibitors that display the requisite combination of picomolar potency and brain bioavailability.

## Methods

### Sex as a biological variable.

All in vivo efficacy studies were conducted with an equal number of male and female mice to capture known sex-differences in GBM survival. All in vivo PK experiments were conducted with only male mice, which is industry standard.

### Compound synthesis, in vitro DMPK, and in vivo PBL studies.

See Supporting Information.

### Primary cell culture.

DI318 was derived from deidentified samples collected from the Cleveland Clinic Brain Tumor and Neuro-Oncology Center. Cell line 3832 was a gift from Duke University, while L0, L1, and L2 CSCs were a gift from the University of Florida. All CSC models were maintained in Neurobasal Complete Medium — NBMc: Neurobasal Medium (Gibco #12349015) + 1X GlutaMax + 1X Sodium Pyruvate + 1X Pen/Strep + 1X Vitamin B27-A Supplement (Life Technology #12587010) + 10 μg/L EGF (R&D #236-EG) + 10 μg/L FGF2 (R&D #4114-TC) — as described previously (24, 83).

### Purification of WDR5.

Recombinant human WDR5 was cloned, expressed, and purified as previously described (37). Briefly, human WDR5 residues Ser22-Cys334 were codon optimized for *E. coli* and cloned into a pET27 plasmid downstream of a SUMO-protease cleavable NT-His6-SUMO fusion. T7 Express *lys*Y/I^q^ *E.coli* cells (New England Biolabs) were transformed with the expression plasmid were grown in Terrific Broth media supplemented with Kanamycin at 37°C with shaking to an optical density λ_600nm_ = 0.6, then reduced to 30°C and induced with 1 mM isopropyl-beta-D-thiogalactoside overnight. Induced cultures were pelleted; resuspended in 1X PBS pH 7.4, 300 mM NaCl, 20 mM imidazole, 5 mM β-Me, and 10% glycerol; and lysed via sonication. Lysates were clarified by centrifugation at 21,000 × *g*, loaded onto an equilibrated NiNTA column, washed with resuspension buffer, and eluted over a gradient of increasing (imidazole). His-SUMO-WDR5 used in the TR-FRET assay was further purified via size exclusion chromatography using 1X PBS pH 7.4, 300 mM NaCl, 5 mM β-Me running buffer, concentrated to 1–2 mg/mL, aliquoted, and froze at –80°C for storage. Tag free WDR5 used for crystallography was dialyzed overnight with SUMO protease and passed over an NiNTA column to remove the His6-SUMO tag. Tag free WDR5 was further purified via size exclusion chromatography using 20 mM HEPES pH 7.0, 250 mM NaCl, and 5 mM DTT; concentrated to 10 mg/mL; aliquoted; and frozen at –80°C for storage.

### WDR5 cocrystallization and data processing.

WDR5 at 10 mg/mL was titrated with 2%–4% v/v inhibitor (10 mM DMSO stock), incubated at room temperature for 1 hour, and cocrystallized by hanging drop vapor diffusion. Crystals formed in previously reported conditions of 0.2M ammonium acetate, 20%–30% PEG 3350, and 0.1M Bis-TRIS, TRIS, or HEPES pH 6–8 within 1–3 days of incubation at 16°C (37). Crystals were cryoprotected with 20% glycerol, looped, and plunge frozen in liquid nitrogen. X-ray diffraction data was collected at the Advanced Photon Source LS-CAT 21-ID-G and 21-ID-F beamlines under cryogenic conditions. Diffraction data was indexed, scaled, and merged using HKL2000 (84). Structures were phased using molecular replacement (Phaser-MR) with PDB: 6UOZ and refined using Phenix Refine with manual rounds of refinement in Coot (85–88). Ligand restraints were generated using ELBOW and final figures generated using PyMOL (89). Final structure statistics can be found in Supplemental Figure 2.

### WDR5 TR-FRET binding assay.

Inhibitor *K*_i_ values were measured using a previously published competitive TR-FRET assay (37). Compounds dissolved at 10 mM in DMSO were serial diluted 5-fold over 10 concentration points and acoustically transferred into 384-well OptiPlates (Revvity) using a Labcyte Echo 550. A 20 μL solution of 1 nM LanthaScreen Elite Tb-anti-His antibody (ThermoFisher), 150 nM “10-mer-Thr-FAM” peptide probe (ARTEVHLRKS-[Ahx-Ahx] [Lys-(5-FAM)]) synthesized by GenScript, and 2 nM recombinant His6-SUMO-WDR5 all diluted in assay buffer (1X PBS pH 7.2, 300 mM NaCl, 0.5 mM TCEP, 0.1% CHAPS) was titrated into each well of the compound-stamped plate for a final 0.1% v/v DMSO. Assay plates were centrifuged at 200 × *g*, covered, and incubated at room temperature for 1 hour prior to spectroscopic measurement (λ_ex_ = 340 nM, λ_em1_ = 495 nm, λ_em2_ = 520 nm) on a BioTek Cytation5 plate reader. The 520/495 TR-FRET ratio was calculated and plotted against the logarithmic inhibitor concentration. For *K*_i_ determination, the sigmoidal displacement curves were fit with a “One site – Fit Ki” analysis using PRISM10 and constraining “HotNM” = 150 and the “HotKdNM” = 2. The reported *K*_i_ values were calculated from at least 3 biological replicates completed in technical quadruplicate.

### WDR5 live cell CETSA.

In total, 500,000 L0 CSCs, grown in suspension as spheroids, were split with Accutase and seeded in 1 mL of NBMc into each well of a 24-well dish. Compounds were prepared in DMSO at 333X concentration and 3 μL (0.3% v/v DMSO) was added to the corresponding wells and incubated for 2 hours. Cells were harvested by centrifugation (5 minutes, 500*g*) into SafeLock tubes (Eppendorf), washed once with 400 μL of PBS, and resuspended in 25 μL of CETSA Buffer (PBS + 1X Roche cOmplete Protease Inhibitor Cocktail + 3X Benzonase). For generation of thermal melting curves, samples were heated for 3 minutes at 45°C–89°C. For isothermal K_d_ determination, samples were heated for 3 minutes at 70°C. One DMSO-treated sample was left unheated (representing 100% abundance i.e., fully bound) and 1 DMSO-treated sample was heated to 70°C (representing 0% abundance i.e., fully unbound). After heating, samples were lysed by 6X freeze-thaw cycles in LN_2_/hot water, rested for 10 minutes at room temperature, and then centrifuged for 30 minutes at > 14,000*g* at 4°C. 20 μL of the supernatant was transferred to a fresh tube into 5 μL of SDS-PAGE Loading Dye, boiled, and separated on 4%–20% Mini-PROTEAN Precast TGX Gels (Biorad). Gels were transferred to PVDF with a Trans-Blot Turbo (Biorad), probed with primary antibodies to WDR5 (1:1000, rb CST #13105) and ACTB (1:3,000 ms #CST3700), and amplified with 1:5,000 LI-COR goat secondary antibodies (IRDYE680RD Anti-Mouse and IRDYE800CW Anti-Rabbit). WDR5 band intensity was quantified on a LI-COR Odyssey imager and normalized from 0% (DMSO heated) to 100% (DMSO unheated), plotted against log[compound], and midpoint K_d_ values were extracted by nonlinear regression in GraphPad PRISM. For CETSA washout experiments, a 10 cm dish with 6 × 10^6^ to 8 × 10^6^ L0 cells was treated for 2 hours with 5 μM compound or DMSO, washed 3 times with 15 mL of PBS (3 min, 400*g*), and split into 10 × 1 mL of fresh NBMc in a 24-well dish. Single wells were taken and prepared for CETSA-WB as described above at different time-points over the course of 21 hours.

### qPCR.

PD CSC lines L0, L1, L2, DI318, and 3832, all grown as spheroids in NBMc, were split with Accutase and 300,000 cells were seeded in 4 mL of NBMc into 6-well dishes (3 wells per condition). In total, 4 μL of a 1,000X compound stock prepared in DMSO (0.1% v/v DMSO) was added and cells were grown at 37°C/5% CO_2_ as standard for 72 hours. Cells were harvested by centrifugation (5 minutes, 500*g*) into 2 mL Eppendorf Tubes and total RNA was purified on spin columns using the RNeasy Plus Kit (Qiagen, includes gDNA removal columns). In total, 2.5 μg of total RNA was treated with ezDNAse (Thermo Fisher) and then reverse transcribed with the SuperScript IV VILO RT Master Mix (Invitrogen). cDNA was diluted 1:100 in nuclease-free water and then transcripts were quantified using the PowerUp SYBR Green Master Mix (Thermo Fisher) on a QuantStudio 3 qPCR machine according to the manufacturer’s recommendations. Primer sets are provided in the Supporting Information. Raw Cq values were normalized per sample to the housekeeper ACTB and then the fold-change relative to DMSO treated cells was calculated as FC = 2^–ΔΔCq^.

### 384w CSC viability assays.

The 333X-1000X compound dilution series were prepared in 100% DMSO by serial-dilution and then stamped by acoustic dispensing (50–150 nL, 0.1%–0.3% DMSO v/v) into 384-well sterile TC-treated white opaque plates using a Labcyte Echo 550. For GI50 determination, compounds were stamped in technical triplicate, while for synergy cross-titrations dose-response matrices were prepared in technical duplicate. Different CSC lines, grown in suspension as spheroids, were split with Accutase and seeded directly on top of the prestamped compounds (50 μL of NBMc and 700 cells/well). The outer 4 rows (A,B and O,P row IDs) and 4 columns (1,2 and 23,24 column IDs) were filled with 80 μL of PBS to minimize evaporation. Cells were incubated with compounds for 7 days at 37°C/5% CO_2_ prior to the addition of 25 μL/well Cell-Titer Glo (Promega) and luminescence quantification on a Revvity EnVision plate reader. Percent viability values were calculated by normalization to DMSO treated cells (100%) or staurosporine treated cells (0%) and GI50 values were extracted by nonlinear regression in GraphPad PRISM. For the cross-titrations of 2 compounds, synergy scores were calculated using the MuSyC webserver (79). For the PiCL screen, the entire library of ~2,100 FDA-approved drugs and chemical probes was screened by CTG as described (except for 5 days) at 1 μM as singletons in the presence of 100 nM C16 against L0 and DI318 CSCs. Any PiCL compound achieving > 80% reduction in cell viability against either CSC line in this primary screen (*n* = 245) was then counter-screened in the presence or absence of 200 nM C16 to identify potential synergistic combinations.

### RNA-seq and data analysis.

L0 and DI318 CSCs were treated were treated for 72 hours with DMSO (0.1% v/v), 200 nM C16, or 200 nM C3TD078 (triplicate for all treatments), and total RNA was purified as described in the qPCR section and then quality controlled for integrity and concentration on a Qubit (Invitrogen). Bulk RNA-seq was performed at MedGenome Inc. using the Illumina Stranded mRNA Library Prep Kit and sequencing at 20M paired-end read-depth on a NovaSeq 6000 (PE100). The raw bulk RNA-seq paired end (R1-R2, 2 × 100 bp) sequencing fastq data was first subjected to the quality check analysis using FastQC (v0.11.9). Then, raw reads were quality filtered utilizing multithreaded fastp tool (v0.23.2) (30423086), majorly implementing deduplication, auto-detect adapter removal, N base removal, Phred quality filtering (≥Q20 is qualified), and unqualified bases percent limit (cut-off to remove reads with unqualified bases > 30%) with other default parameters. Further, quality filtered reads were subjected to the alignment employing Rsubread (v2.16.1) align function with human genome reference build GRCh38 (90, 91). The mapped binary alignment map (BAM) files were indexed using samtools (v1.16.1) (92), and gene-wise read counts were summarized applying featureCounts function from Rsubread, where inbuilt hg38 annotation was used and duplicated reads were ignored. Furthermore, gene symbols were obtained using org.Hs.eg.db (v3.15.0) annotation package operating on ENTREZIDs from count table. Respectively, edgeR (v3.38.4) and limma-voom (v3.52.4) packages were employed to accomplish differential transcriptional regulation analysis (93–95). Briefly, constructed DGEList object (DGEList function), design matrix (model.design), and contrast matrix (makeContrasts) was defined, filtered low counts (filterByExpr), and trimmed mean of M-values (TMM) normalization (calcNormFactors) was done. Later, log_2_ counts per million reads (logCPM) transformation, model fitting and differential expression analysis was performed using limma-voom (voomWithQualityWeights, lmFit, contrasts.fit), empirical Bayes moderated t-statistics (eBayes), and TREAT method (treat, decideTests, topTreat) considering log fold-change (logFC) (0.5) threshold. Genes with |log_2_FC| > 0.5 and FDR adjusted *P* value cutoff (≤0.01) were considered as differentially expressed genes (DEGs). Gene ontology (GO) and pathway enrichment analysis was accomplished implementing ShinyGO (v0.77) (96). DEGs volcano plots were depicted utilizing EnhancedVolcano (v1.16.0) package. Standard GSEA was performed using GenePattern against the set of genes measured by Howard et al. to be downregulated (*n* = 191; log_2_FC < –0.5, *P* < 0.01) by C16 treatment in MV4:11 cells (33, 68, 97).

### Extreme Limiting Dilution Assay (ELDA).

L0 and DI318 CSCs were split with Accutase as single cells into NBMc. In total, 200 μL of CSCs were seeded into each well of a 96-well TC treated plate (*n* = 10 well per density) at 50, 25, 13, 6, 3, and 1.5 cells/well. One full plate was treated with either DMSO (0.2% v:v) or the indicated doses of C3TD078. Spheroids were allowed to grow for 14 days at 37°C/ 5% CO_2_ followed by manual classification of each well for the binary presence or absence of sphere(s) (independently assessed by 2 scientists). The stem cell frequency was quantified and treatments were compared with DMSO as described by Hu and Smyth (98).

### In situ brain perfusion technique.

To evaluate the permeability across the blood-brain barrier of WDR5 inhibitors in the absence of hepatic clearance, we utilized the in situ brain perfusion technique. Mice were anesthetized via i.p. injection of 100 mg/kg ketamine and 80 mg/kg xylazine. The peritoneal cavity was exposed, the descending aorta was clamped, and the right atrium was cut to prevent venous return to the heart. A 28G butterfly winged infusion catheter (Terumo Medical Care Solutions) was inserted into the left ventricle and mice were perfused with a modified physiological buffer containing 10 μM WDR5 inhibitor (99). Perfusion fluid pumped through the left cardiac ventricle and maintained at a constant rate 2.5 mL/min using a Pump 11 Elite Syringe Pump (704500, Harvard Apparatus). To determine brain uptake of WDR5 inhibitors, mice were perfused for 120 seconds. The experiment was concluded by decapitation of the mouse. Following termination, the brain was rapidly removed and placed on ice. Inhibitor concentration in both brain and blood was determined by liquid chromatography tandem mass spectroscopy (Q2 Solutions). The unidirectional uptake transfer constant (K_in_) was calculated by the following relationship (100):

(Equation 1)
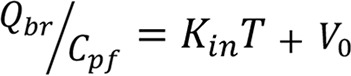


Where Q_br_ is the concentration of WDR5 inhibitor in brain (ng/g) corrected for residual inhibitor remaining in cerebrovasculature at the end of the perfusion time frame, C_pf_ is the perfusion fluid concentration of WDR5 inhibitor (ng/mL), T is the time length of the perfusion experiment, and V_0_ is the vascular volume of the WDR5 inhibitor (mL/g). For these experiments, an average value of 0.012 mL/g was used for vascular volume (101, 102).

### Long-term intracranial L0 xenografti.

Intracranial xenografts were conducted as previously described (103, 104). Briefly, L0 cells were intracranial injected into 6–12 week old NSG mice. Once fully anesthetized, mice were secured into a stereotaxic apparatus and 20,000 L0 Cells were injected into the left hemisphere 0.5 mm rostral and 1.8 mm lateral to the b4regma with 3.5 mm depth from the scalp. One week after tumor implantation, animals were treated with for 80 days with 30 mg/kg once a day i.p.C3TD343 formulated in 20% BCD. Animals were monitored over time for the presentation of neurological and behavioral symptoms at which time the animals were humanely euthanized. Survival analysis was completed using PRISM 9.1.2 and comparison were made using Log-Rank (Mantel-Cox) test.

### Statistics.

Relevant statistical tests and number of biological replicates are described in the corresponding figure legends. Statistical tests were always corrected for multiple comparisons and significance was assessed at *P* < 0.05 for biological experiments or *P* < 0.01 for differentially expressed genes during RNAseq. Technical outliers were excluded if > 5× standard deviations away from the mean of the group. No biological replicates were excluded from any experiments — all data were presented.

### Study approval.

All animal procedures were performed in accordance with the guidelines and protocols approved by the IACUC at the Cleveland Clinic and by the Walter and Eliza Hall Institute Animal Ethics Committee. NSG (NOD.Cg-PrkdcscidIl2rgtm1Wjl/SzJ) mice were obtained from the Biological Research Unit (BRU) at Cleveland Clinic Research.

### Data availability.

RNA-seq data associated with this manuscript have been deposited to GEO (GSE304999). Crystallographic maps and coordinates were deposited to the Protein Data Bank (PDB: 9NCW, 9NCT, 9NCV). All other data are provided in the main text and/or the associated Supporting Data Values file.

## Author contributions

JAC designed and conducted the cellular experiments and wrote the manuscript. JAC, CMG, and AKG prepared figures. SAS conducted the in vivo perfusion experiments, advised on all pharmacokinetics, and conducted intracranial xenograft. SRM, SHH, JM, DS, JA, and SRS designed and synthesized the reported compounds. AKG and FC assisted with bioinformatic analyses. ARS, GB, JHR, TN, NR, TR, and NSW assisted with the cellular experiments. PP, EGR, AAL, and AR performed protein purification, x-ray crystallography, and biochemical assays. ARS and GB conducted the flank xenograft. CMG solved the reported crystal structures. SAS, ARS, GB, CMG, NSW, JDL, and SRS provided editorial guidance for the manuscript, which was approved in its final form by all authors. JA, CGH, JDL, and SRS secured funding and provided supervision for the project.

## Conflict of interest

The authors have declared that no conflict of interest exist.

## Funding support

The following organizations provided funding support:

- Falk Medical Research Trust Catalyst Awards Program
- Cleveland Clinic Research.

## Supplementary Material

Supplemental data

Unedited blot and gel images

Supporting data values

## Figures and Tables

**Table 1:**
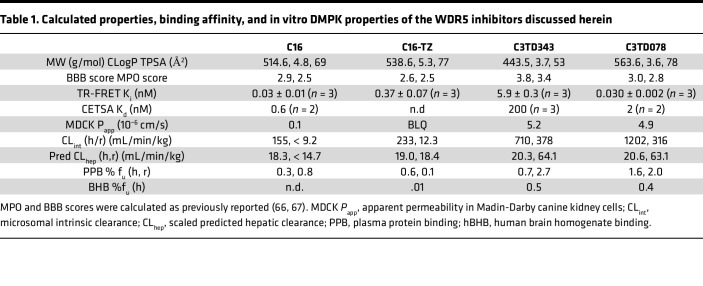
Calculated properties, binding affinity, and in vitro DMPK properties of the WDR5 inhibitors discussed herein

**Table 2:**
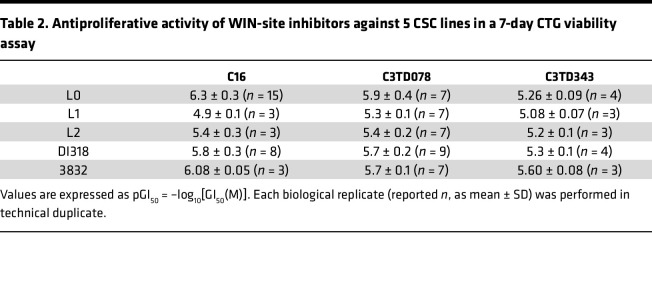
Antiproliferative activity of WIN-site inhibitors against 5 CSC lines in a 7-day CTG viability assay

## Version 1 05/05/2026

In-Press Preview

## Version 2 06/22/2026

Electronic publication
