# Supplementary material for: Development and characterization of triazole-based WDR5 inhibitors for the treatment of glioblastoma
Source: JCI Insight. 2026 May 5;11(12):e198298. doi: 10.1172/jci.insight.198298 (PMC13313556; doi:10.1172/jci.insight.198298)
Supplement: Supplemental data [file jciinsight-11-198298-s300.pdf]

SUPPORTING INFORMATION

Development and Characterization of Triazole-Based WDR5 Inhibitors for the Treatment of Glioblastoma

Jesse A. Coker[1,2], Steven R. Martinez[2], Sang Hoon Han[2,3], Anthony R. Sloan[4,5], Amit Kumar Gupta[6,7], George Bukenya[4], Paul Polzer[2], James H. Ramos[4], Emma Rico[2], Annabella Rico[2], A. Abigail Lindsey[2], Tanvi Navadgi[4], Natalie Reitz[4], Todd Romigh[2], Jonathan Macdonald[2], Dhiraj Sonawane[2], Christopher M. Goins[1,2], Chris G. Hubert[5,8], Nancy S. Wang[2], Feixiong Cheng[1,5,6,7], Joseph Alvarado[1,2], Samuel A. Sprowls[9,10], Justin D. Lathia[4,5,11]*, Shaun R. Stauffer[1,2]*

TABLE OF CONTENTS

- RT-qPCR Primer Sets
- Supplemental DMPK Methods
- Compound Synthesis and Characterization
- Supplemental Figure 1: Plasma:blood level (PBL) study for **C16**
- Supplemental Figure 2: Data collection and refinement statistics for novel WDR5 co-crystal structures
- Supplemental Figure 3: X-ray crystal structures of WDR5 in complex with triazole based inhibitors with differing S2, S4, and S7 pieces
- Supplemental Figure 4: Permeability of **C16** and **C3TD343** in MDR1-MDCK cells
- Supplemental Figure 5: Volcano plots and biological processes GO enrichment for RNAseq study of **C16** and **C3TD879** in L0 and DI318 CSCs
- Supplemental Figure 6: Core set of WIN-site regulated genes in CSCs
- Supplemental Figure 7: Enriched GO terms and pathways in CSC RNAseq studies
- Supplemental Figure 8: CSC viability assays with **C16** and **C3TD879** in combination with radiation
- Supplemental Figure 9: Expression of *PYGB* in CSCs as assessed by RT-qPCR
- Supplemental Figure 10: L0 CETSA washout for **C16**
- Supplemental Figure 11: Chemical structure and *in vitro* profile of **C3TD424**
- Supplemental Figure 12: Phospholipidosis assay in A549 cells with **C16** and **C3TD879**
- Supplemental Figure 13: Cross-titration experiments with **C16** and **C3TD078** in combination with rationally selected anti-cancer drugs including Venetoclax
- Supplemental Figure 14: Protein expression of WDR5, ATAD2, and p53 in CSCs
  Supplemental Figure 15: Cross-titration experiments with **C3TD078** and the ATAD2 bromodomain inhibitor **GSK-8814** in five different CSC models.
- Supplemental Figure 16: Expression of WDR5-target genes in CSCs treated with **BAY-850.**
- Supplemental Figure 17: Results from DI318 flank xenograft model with **C3TD078**.

PRIMER SETS

TRANSCRIPT	FORWARD PRIMER (5'-3')	REVERSE PRIMER (5'-3')
ACTB	CTGGAACGGTGAAGGTGACA	AAGGGACTTCCTGTAACAATGCA
RPS24	GACACCGTAACTATCCGCACT	TCTTAGGCACTGTCGCCTTC
RPL14	GTCTCCTTTGGACCTCATGC	ATGGCCTGTCTCCTCACTTG
CKMT1	ATATGACCCCCGGACAATGAA	CTTCGGCCAGTTCTGACTCT
PYGL	CAGCCTATGGATACGGCATTC	CGGTGTTGGTGTGTTCTACTTT
PYGB	AGGTGCGGAAGAGCTTCAAC	TCGCGCTCGTAGTAGTGCT
SALL2	GGCTTGCCTTATGGTATGTCCG	TGGCACTGAGTGCTGTTGTGGA
NID2	GCCCGGTCAAAGAGGATTCA	TGCGCACTCACAGGTGTAAT

SUPPLEMENTAL DMPK METHODS

In Vitro Tier 1 DMPK

All *in vitro* DMPK screening assays were performed at Q2 Solutions, Inc. (Indianapolis, IN, USA). ***Microsomal Intrinsic Clearance:*** Test compounds (1 µM) were incubated with 1 mg/mL of pooled liver microsomes in phosphate buffer at 37 °C. Samples were taken at 0, 5, 10, 15, 30 and 45 min and analyzed by LC-MS. Testosterone was used as the positive control. Two replicates were obtained and average CL_{int} values are reported. ***Plasma Protein and Brain Homogenate Binding:*** Plasma protein binding assays in human and rat plasma were performed using equilibrium dialysis method and a fast gradient elution LC-MS/MS to estimate the percent of compound which binds to protein over a 4.5 hour incubation period at 37 ºC, while undergoing orbital shaking. Assay was performed using a HT dialysis micro equilibrium device using dialysis membrane strips. A time 0 sample was taken after protein matrix is created and samples were taken from

both the protein side and buffer side of the membrane after incubation and the parent compound was quantified. Brain homogenate binding was performed similarly as above using human brain homogenate, equilibrium dialysis, and LC-MS/MS methods. Fraction unbound is calculated by dividing the concentration of the buffer side by the concentration of the matrix side (plasma or brain). ***MDCK Passive Permeability:*** MDCK (Madin-Darby canine kidney) assay plates were seeded 3-4 days prior to running the assay. PET 24-well plates were seeded at a cell density of 0.875 x 10^5 cells/well in a 250 µL apical well volume (3.52 x 10^5 cells/mL) with a 1.0 mL volume of growth medium to the 24-well basolateral wells. The basolateral wells were rinsed once and the apical wells were rinsed twice with HEPES Buffer Saline (HBSS), and fresh HBSS was added to the assay plate in a 250 µL apical well volume and a 1.0 mL basolateral well volume. The apical chamber was incubated with Pgp inhibitor LSN335984 for 36 minutes at 37 ºC and then replaced with test article and incubated for a 5 minute and 60 minute interval. Acetonitrile was added to each well of the basal chamber of the assay plate, which is mixed with the existing buffer and transferred to the LCMS plate for analysis using a SCIEX API 4000/5000 instrument. Dexamethasone and Atenolol were utilized as controls. Compound transport was measured in the absorptive direction and expressed as the percentage transport over the incubation period.

In Vivo Intraperitoneal Plasma:Brain Level (PBL) Studies

In vivo PBL studies for **C16** and **CCF343** were performed by the Rat Metabolic Physiology Core (RMPC) at the Vanderbilt University Medical Center (VUMC) Metabolic Physiology Shared Resource Core (MPSR). The studies were performed according to guidelines

approved by the Institutional Animal Care and Use Committee (IACUC) of VUMC following the guidance of the Association for Assessment and Accreditation of Laboratory Animal Care (AAALAC). Male CD-1 mice (Charles River Laboratories, Wilmington, MA) were overnight fasted on the evening prior to study. On the morning of study mice were weighed and allowed to acclimate to the room for at least 30 minutes prior to dosing. Food was returned 3 hr after intraperitoneal dosing. At time zero IP of test article was given (20% β-HPCD, 1-10 mg/mL with addition of 1N HCl per molar equivalent of test article, N=8 mice, two per timepoint). At 0.5, 1, 3, and 6 h post-dose, mice were placed into a plane of anesthesia using Isoflurane. A terminal blood sample was collected via cardiac puncture followed by immediate euthanasia and brain collection. Brain was washed with cold PBS or Saline, blotted dry on a piece of gauze, weighed, and flash frozen in liquid nitrogen. Whole blood was centrifuged at 5000 x *g* for 5 minutes and plasma was removed into a fresh tube for storage. All samples were stored at -80 ºC until shipment on dry ice for bioanalysis at Q2 Solutions. For **C3TD078**, PBL studies were conducted at Medicilon Inc. DMPK (CRO, Shanghai) using a similar protocol as above using modified timepoints (5 min, 15 min, 30 min, 1, 3, and 8 h), male ICR mice, and a formulation of 10 mg/mL of test article in 5% NMP + 10% Solutol + 85% (20% HP-β-cyclodextrin). The blood was taken via submandibular vein or other suitable vein, 0.03 mL/time point. Samples were placed in tubes containing K2-EDTA and stored on ice until centrifuged. The blood samples were centrifuged at 6800 x *g* for 6 minutes at 2-8°C within 1h after collection and stored frozen at -80°C. After blood sample collection, the brain was removed, rinsed with saline, dried with filter paper, placed into labeled EP tubes (1 tube/tissue/animal/time point, and frozen at -80°C prior to bioanalysis. The analytical

results were validated using a calibration curve to lower limit of quantification of 2 ng/mL for parent analyte with tolbutamide as an internal standard.

COMPOUND SYNTHESIS AND CHARACTERIZATION

Dihydroisoquinoline-based WDR5 inhibitors **C16** was synthesized and characterized as described previously(1). Triazole-based WDR5 inhibitors were synthesized according to **Scheme 1**. Details regarding synthetic method development will be reported elsewhere in due course. Briefly, starting from readily available methyl 3,5-dibromo-4-methylbenzoate **1**, the dibromo-1,5-disubstitued-1,2,3-tiazole key intermediate was achieved in six-steps. A novel one-pot tandem Suzuki Cross-Coupling and subsequent intramolecular C-H activation was utilized to form the 1,4,5-trisubstituted-1,2,3-triazole scaffold, the average yield for tricyclic formation was 50%. A subsequent three-step sequence involving deprotection, activation, and displacement yielded the fully functionalized target with the S2 warhead installed. An exemplary synthesis of **C16-TZ** is detailed below.

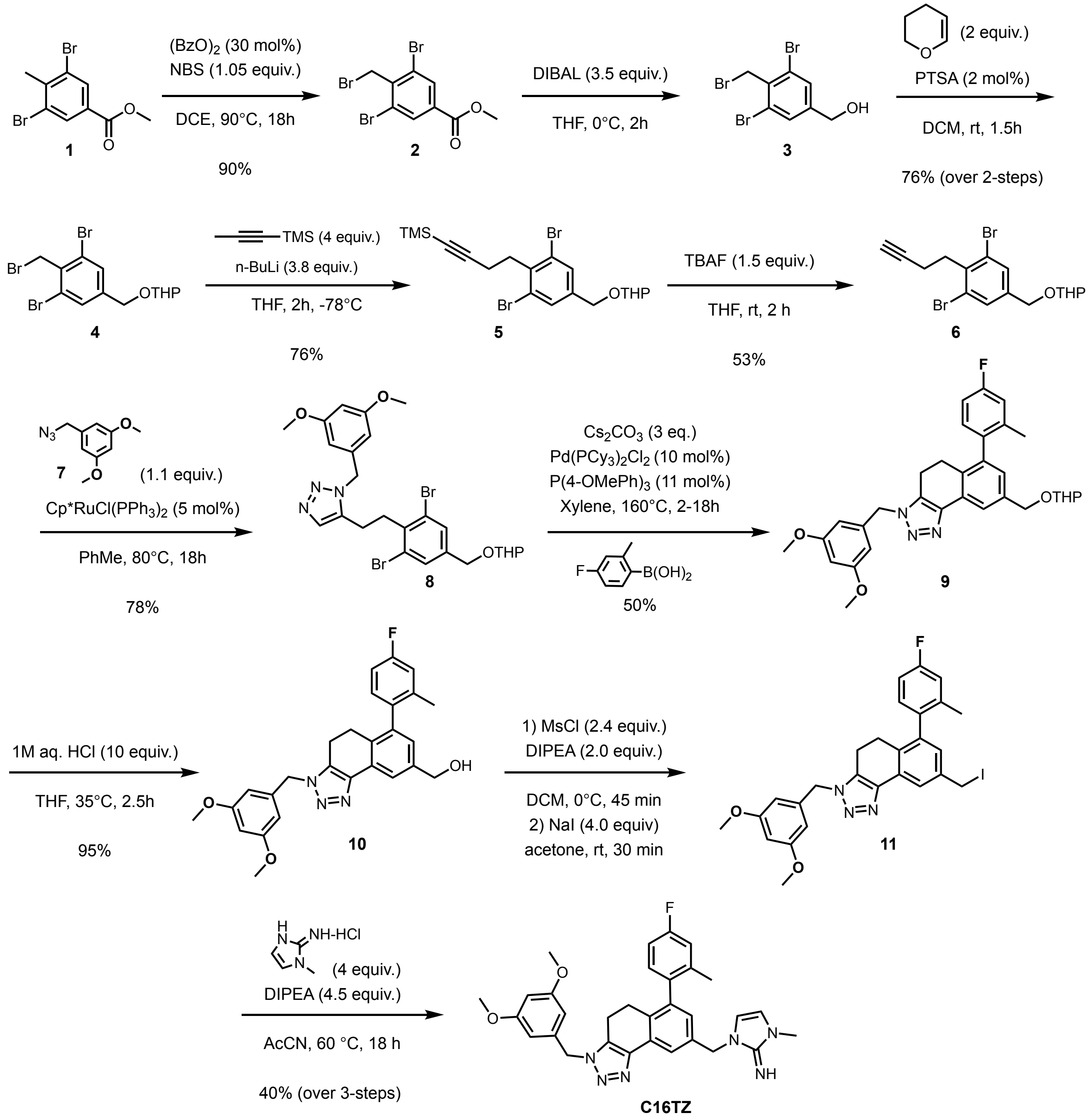

Scheme 1. Synthesis of triazole-based WDR5 inhibitor **C16-TZ**.

1-((3-(3,5-Dimethoxybenzyl)-6-(1-ethyl-3-(trifluoromethyl)-1H-pyrazol-4-yl)-4,5-dihydro-3H-naphtho[1,2-d][1,2,3]triazol-8-yl)methyl)-3-methyl-1,3-dihydro-2H-imidazol-2-imine (**C16-TZ**):

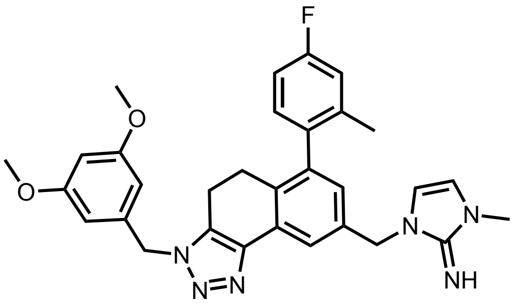

Step 1. Preparation of **methyl 3,5-dibromo-4-(bromomethyl)benzoate (2).** To a 500 mL round bottom flask with a stir bar, the following were charged in the following order: methyl 3,5-dibromo-4-methyl-benzoate (**1**, 1.0 equiv., 24 g, 77.93 mmol), *N*-bromosuccinimide (1.05 equiv., 14.56 g, 81.8 mmol), benzoyl peroxide (0.3 equiv., 5.66 g, 23.3 mmol) and DCE (243.5 mL). The reaction mixture was heated to 90 °C and allowed to stir 18 h. The reaction mixture was cooled to room temperature and sat. NH_4Cl (50 mL) added. The organic layer was separated, and the aq. layer was extracted with DCM (3 x 40 mL). The combined organic layers were washed with H_2O (2 x 25 mL), sat. brine (50 mL), and dried over Na_2SO_4. The solids were filtered off, washed with DCM (2 x 25 mL) and the filtrate was concentrated under vacuum to give ~27 grams of **2** as a white solid (90%). The crude solid was used without purification: ^1H NMR (400 MHz, $CDCl_3$) δ 8.19 (s, 2H), 4.82 (s, 2H), 3.93 (s, 3H).

Step 2. Preparation of (3,5-dibromo-4-(bromomethyl)phenyl)methanol (**3**). To a 500 mL round bottom flask with a stir bar, **2** (methyl 3,5-dibromo-4-(bromomethyl)benzoate, 1.0 equiv., 10.5 g, 27.14 mmol) was dissolved with THF (86 mL) and the solution placed under argon atmosphere. The solution was cooled to 0 °C and 1M diisobutylaluminium hydride in THF (3.7 equiv., 100 mL) was added dropwise to the solution. The reaction mixture was stirred at 0 °C for 2.5 h. The reaction mixture was diluted with Et_2O (100 mL),

quenched at 0 °C by careful addition of 3M aq. NaOH (5 mL) followed by H_2O (10 mL) dropwise (highly exothermic). The quenched solution was warmed to room temperature, stirred for 15 min, charged with $MgSO_4$ (20 g) and then stirred for 15 min. The solids were filtered through a pad of Celite; the solids were mixed in EtOAc and filtered again. The collected filtrates were concentrated under vacuum to give a white solid upon further drying overnight (27.2 g) to give crude product **3** (>90% purity): ^{1}H NMR (400 MHz, DMSO) δ 7.63 (s, 2H), 5.46 (s, 1H), 4.82 (s, 2H), 4.49 (s, 2H).

Step 3. Preparation of 2-((3,5-dibromo-4-(bromomethyl)benzyl)oxy)tetrahydro-2*H*-pyran (**4**). To a 200 mL round bottom flask with a stir bar, the following were charged in the following order: **3** (3,5-dibromo-4-(bromomethyl)phenyl]methanol, 1.0 equiv., 9.74 g, 27.1 mmol), DCM (136 mL), 3,4-dihydro-2*H*-pyran (2.0 equiv., 4.95 mL, 54.3 mmol), and *p*-toluene sulfonic acid monohydrate (0.02 equiv., 103 mg, 0.54 mmol). The reaction mixture was stirred at room temperature for 1 hour. The reaction mixture was quenched with sat. $NaHCO_3$ (10 mL) and vigorously stirred for 5 min. Sat. brine (15 mL) was then added and vigorously stirred for 5 mins. The organic layer was separated and dried over Na_2SO_4. The solids were filtered off, washed with DCM (2 x 25 mL) and the filtrate was concentrated under vacuum to give a crude yellow oil. The crude oil was purified by automated flash chromatography using a 330 g silica column and a gradient of 0-20% EtOAc in hexanes. The desired fractions were pooled and concentrated under vacuum to give the title product **4** as a clear yellow oil (9.14 g, 20.6 mmol, 76% two steps): ^{1}H NMR (400 MHz, $CDCl_3$) δ 7.56 (s, 2H), 4.93 (s, 2H), 4.72 – 4.67 (m, 2H), 3.86 (ddd, *J* = 11.3, 8.7, 3.4 Hz, 1H), 3.56 (ddd, *J* = 10.9, 5.4, 2.9 Hz, 1H), 1.92 – 1.54 (m, 7H).

Step 4. Preparation of (4-(2,6-dibromo-4-(((tetrahydro-2*H*-pyran-2-yl)oxy)methyl)phenyl)but-1-yn-1-yl)trimethylsilane (**5**). To a 200 mL round bottom flask with a stir bar under Argon, THF (39.1 mL) and trimethyl(prop-1-ynyl)silane (4.0 equiv., 3.48 mL, 23.5 mmol) were charged. The solution was cooled to -78 °C, 2.5M *n*-BuLi in THF (3.8 equiv., 8.92 mL, 22.3 mmol) was charged dropwise; the reaction mixture was stirred for 2 h at -78 °C. A solution of **4** (2-[[3,5-dibromo-4-(bromomethyl)phenyl]methoxy]tetrahydropyran) from step 3 (1.0 equiv., 2.6 g, 5.87 mmol) in THF (19.57 mL) was charged dropwise; the reaction mixture was stirred for 2h at -78°C. At -78 °C, the reaction was quenched with MeOH (20 equiv. 4.75 mL) and the solution was allowed to warm. At 2-8 °C, EtOAc (60 mL), sat. NH_4Cl (20 mL), H_2O (10 mL) were charged and the mixture was warmed to room temperature. The organic layer was separated, and the aq. layer was extracted with EtOAc (30 mL). The combined organic layers were washed with sat. brine (20 mL), dried over Na_2SO_4, the solids filtered off, then washed with EtOAc (2 x 15 mL). The filtrate was then concentrated under vacuum to give a crude oil. The crude oil was purified by automated flash chromatography using an equilibrated 80 g silica column with 0-20% EtOAc in hexanes gradient. The desired fractions were pooled and concentrated under vacuum to give **5** as a clear yellow oil (2.11 g,4.45 mmol, 76% yield): ^{1}H NMR (400 MHz, $CDCl_3$) δ 7.50 (s, 2H), 4.72 – 4.63 (m, 2H), 4.39 (d, *J* = 12.5 Hz, 1H), 3.92 – 3.82 (m, 1H), 3.60 – 3.50 (m, 1H), 3.22 (t, *J* = 8.0 Hz, 2H), 2.51 (dd, *J* = 16.1, 8.0 Hz, 2H), 1.86 (qd, *J* = 11.6, 9.3, 2.6 Hz, 1H), 1.80 – 1.70 (m, 1H), 1.70 – 1.47 (m, 5H), 0.15 (s, 9H).

Step 5. Preparation of 2-[(3,5-dibromo-4-but-3-ynyl-phenyl)methoxy]tetrahydropyran (**6**). To a 100-mL round bottom flask with a stir bar, intermediate **5** (4-[2,6-dibromo-4-(tetrahydropyran-2-yloxymethyl)phenyl]but-1-ynyl-trimethyl-silane, 1.0 equiv., 8 g, 16.9 mmol) was dissolved with THF (56 mL). A solution of TBAF (1M in THF, 1.5 equiv., 25.3 mL, 25.3 mmol) was charged at 23 °C. The reaction was stirred for 2 h at 23 °C. EtOAc (125 mL), sat. NH_4Cl (40 mL) and H_2O (20 mL) were charged and allowed to stirred for 10 min. The organic layer was separated, and the aq. layer was extracted with EtOAc (2 x 75 mL). The combined organic layers were washed with brine, dried over Na_2SO_4, the solids were filtered off and washed with EtOAc (2 x 20 mL). The filtrate was concentrated under vacuum to give a crude yellow oil. The crude oil was purified by automated flash chromatography using an equilibrated 330 g silica column and a 0-20% EtOAc in hexanes gradient. The desired fractions were pooled and concentrated under vacuum to give the desired product **6** as a clear yellow oil (5.5 g, 13.68 mmol, 81% yield): ^{1}H NMR (400 MHz, CDCl3) δ 7.51 (s, 2H), 4.75 – 4.65 (m, 2H), 4.40 (d, *J* = 12.5 Hz, 1H), 3.92 – 3.83 (m, 1H), 3.61 – 3.51 (m, 1H), 3.31 – 3.20 (m, 1H), 2.56 (s, 1H), 2.47 (ddd, *J* = 9.8, 6.8, 2.6 Hz, 1H), 2.02 (t, *J* = 2.6 Hz, 1H), 1.97 – 1.56 (m, 7H).

Step 6. Preparation of 5-(2,6-Dibromo-4-(((tetrahydro-2*H*-pyran-2-yl)oxy)methyl)phenethyl)-1-(3,5-dimethoxybenzyl)-1*H*-1,2,3-triazole (**8**). To a 100 mL round bottom flask with a stir bar, **6** (2-[(3,5-dibromo-4-but-3-ynyl-phenyl)methoxy]tetrahydropyran, 1.0 equiv., 5.g, 12.43mmol), 1-(azidomethyl)-3,5-dimethoxy-benzene (**7**, 1.2 equiv., 2.9 g, 14.9 mmol), toluene (51.8 mL), chloro(pentamethylcyclopentadienyl)bis(triphenylphosphine)ruthenium(II) (0.045 equiv.,

445.6 mg, 0.56 mmol) were charged. The mixture was sparged with argon for 20 min. The reaction was heated to 80 °C for 18 h. The reaction was cooled to room temp and the solids were filtered off and rinsed with EtOAc; the filtrate was concentrated under vacuum. The crude oil was diluted with minimal CH_2Cl_2, dried loaded and then purified by automated flash chromatography using a 120 g silica column (0-100% EtOAc:hexanes; product eluted at ~70% EtOAc). The desired fractions were pooled and concentrated to give **8** as a viscous orange oil (5 g, 8.4 mmol, 68% yield): ^{1}H NMR (400 MHz, $CDCl_3$) δ 7.55 (s, 1H), 7.49 (s, 2H), 6.34 (t, *J* = 2.2 Hz, 1H), 6.27 (d, *J* = 2.3 Hz, 2H), 5.46 (s, 2H), 4.70 – 4.64 (m, 2H), 4.38 (d, *J* = 12.6 Hz, 1H), 3.85 (ddd, *J* = 11.2, 8.4, 3.0 Hz, 1H), 3.71 (d, *J* = 1.9 Hz, 6H), 3.58 – 3.50 (m, 1H), 3.21 – 3.13 (m, 2H), 2.83 – 2.75 (m, 2H), 1.89 – 1.47 (m, 6H). ^{13}C NMR (101 MHz, $CDCl_3$) δ 161.30, 140.49, 137.35, 137.26, 136.12, 133.10, 131.41, 124.96, 105.07, 100.11, 98.17, 66.93, 62.30, 55.45, 51.73, 35.82, 30.48, 25.40, 21.51, 19.32.

Step 7 and 8. Preparation of **(3-(3,5-dimethoxybenzyl)-6-(4-fluoro-2-methylphenyl)-4,5-dihydro-3H-naphtho[1,2-d][1,2,3]triazol-8-yl)methanol (10).** To a 100 mL round bottom flask a stir bar, 5-[2-[2,6-dibromo-4-(tetrahydropyran-2-yloxymethyl)phenyl]ethyl]-1-[(3,5-dimethoxyphenyl)methyl]triazole (1.0 equiv., 1.0 g, 1.68 mmol), 4-fluoro-2-methylphenyl)boronic acid (2 equiv., 0.52 g, 3.36 mmol), tris(4-methoxyphenyl)phosphane (0.11 equiv., 65 mg, 0.18 mmol), dichloropalladium;tricyclohexylphosphane (0.10 equiv., 124 mg, 0.10 mmol), Cs_2CO_3 (4.0 equiv., 2.19 g, 6.7 mmol) and *m*-xylene (28 mL) were charged. The mixture was sparged with argon for 20 min. The reaction mixture was refluxed to 150 °C and allowed to stir

vigorously (1500 rpm). The reaction was monitored by LC-M. After 3 h, the reaction was cooled to room temp and diluted with EtOAc (150 mL) and H2O (40 mL). The aqueous layer was extracted with EtOAc (3 x 50 mL). The combined organic layers dried over Na2SO4, the solids were filtered and rinsed with EtOAc. The filtrate and combined organic layers were concentrated and dried under vacuum. The crude oil was dissolved with THF (15 mL) and then 1M aq. HCl (10 equiv., 16.8 mL, 16.8 mmol) was charged. The reaction was heated to 35 °C for 2.5 h. The reaction mixture was cooled to room temp and diluted with EtOAc (35 mL) and sat. bicarb. was charged, dropwise (~6 mL), until the pH was basic. The aq. layer was extracted with EtOAc (2 x 15 mL). The combined organic layer was washed with brine, dried over Na_2SO_4, the solids were filtered off and rinsed with EtOAc; the filtrate was concentrated under vacuum. The crude oil was dissolved with minimal DCM and then purified by automated flash chromatography (0-100% EtOAc/hexanes, product elutes at 100% EtOAc). The desired fractions were pooled and concentrated under vacuum. The resulting foam was dissolved in minimal THF and while stirring triturated with addition of hexanes (~40 v/v% THF/hexanes) to afford a white solid. The solid was filtered and rinsed using 40% THF:hexanes and then dried under vacuum to afford the desired carbinol product as a while solid (350 mg, 45% yield): Purity ≥95% by LCMS (210, 254 nm); t_R = 1.69 min, *m/z* = 460.20 $[M+H]^+$, ^{1}H NMR (400 MHz, $CDCl_3$) δ 7.99 (s, 1H), 7.35 (s, 1H), 7.17 (s, 1H), 6.39 (s, 1H), 6.35 (s, 2H), 5.46 (s, 2H), 4.74 (s, 2H), 4.26 (q, *J* = 7.4 Hz, 2H), 3.74 (s, 6H), 2.80 (t, *J* = 7.6 Hz, 2H), 2.68 (t, *J* = 7.4 Hz, 2H), 1.56 (t, *J* = 7.4 Hz, 3H). ^{19}F NMR (376 MHz, $CDCl_3$) δ -60.21.

Step 9. Preparation of 3-(3,5-dimethoxybenzyl)-6-(4-fluoro-2-methylphenyl)-8-(iodomethyl)-4,5-dihydro-3H-naphtho[1,2-*d*][1,2,3]triazole. To a 20 mL vial with a stir bar was charged (3-(3,5-dimethoxybenzyl)-6-(4-fluoro-2-methylphenyl)-4,5-dihydro-3H-naphtho[1,2-d][1,2,3]triazol-8-yl)methanol (1.0 equiv., 230 mg, 0.5 mmol), CH_2Cl_2 (5 mL) and DIPEA (2.0 equiv., 0.17 mL, 1.0 mmol). The solution was cooled to 0°C, methanesulfonyl chloride (0.05 mL, 0.6 mmol) was added to the solution and stirred for 20 mins. Water (10 mL) was added to the reaction mixture at 0°C and the aq. layer was extracted with DCM (3 x 15 mL). The combined organic layers were dried over Na_2SO_4 and concentrated under vacuo. The crude oil, (3-(3,5-dimethoxybenzyl)-6-(4-fluoro-2-methylphenyl)-4,5-dihydro-3H-naphtho[1,2-*d*][1,2,3]triazol-8-yl)methyl methanesulfonate was dissolved with acetone (10 mL). To this solution, sodium iodide (4.0 equiv., 60 mg, 0.4 mmol) was added and allowed to stir at room temperature for 30 mins. Sat. brine (7 mL) was added to the reaction mixture and extracted with EtOAc (2 x 10 mL). The combined organic layers were dried over Na_2SO_4 and concentrated under vacuo. The crude was dissolved with minimal DCM:THF (1:1) and purified by automated flash chromatography (0-45% EtOAc/hexanes). The desired fractions were pooled and concentrated under vacuo, to give 61 mg the desired product as a yellow oil (21% overall yield, two steps): Purity >95% by LCMS (215, 254 nm); t_R = 2.45 min, *m/z* = 571.09 $[M+H]^+$

Step 10. Preparation of **C16-TZ 1-((3-(3,5-Dimethoxybenzyl)-6-(1-ethyl-3-(trifluoromethyl)-1*H*-pyrazol-4-yl)-4,5-dihydro-3*H*-naphtho[1,2-d][1,2,3]triazol-8-yl)methyl)-3-methyl-1,3-dihydro-2*H*-imidazol-2-imine.** To a 1-dram vial with a stir bar,

3-(3,5-dimethoxybenzyl)-6-(4-fluoro-2-methylphenyl)-8-(iodomethyl)-4,5-dihydro-3H-naphtho[1,2-d][1,2,3]triazole (1.0 equiv., 28.5 mg, 0.05 mmol), 3-methyl-1H-imidazol-2-imine hydrochloride (20 mg, 0.15 mmol, 3 equiv.), acetonitrile (0.30 mL) and DIPEA (0.035 mL, 0.20 mmol, 4 equiv.) were charged. The mixture was warmed to 60 °C and stirred for 18 h. The reaction mixture was cooled to room temp and the solids were filtered off. The filtrate was concentrated under vacuum. The crude oil was diluted by minimal DMSO and purified by reverse phase. The desired fractions were pooled and concentrated under positive nitrogen stream. The residue was diluted in EtOAc and washed with sat. K_2CO_3 to remove TFA. The organic layers were washed with brine, filtered from Na_2SO_4, and dried under vacuum to afford the title product as a white solid (13 mg, 48%): Purity ≥95% by LCMS (210, 254 nm); t_R = 2.048 min, *m/z* = 539.23 $[M+H]^+$; ^1H NMR (400 MHz, MeOD) δ 7.73 (d, *J* = 2.0 Hz, 1H), 7.12 – 7.00 (m, 2H), 7.00 – 6.91 (m, 2H), 6.70 – 6.56 (m, 2H), 6.46 – 6.40 (m, 1H), 6.37 (d, *J* = 2.2 Hz, 2H), 5.52 (s, 2H), 5.03 – 4.95 (m, 2H), 3.73 (s, 6H), 3.39 – 3.32 (m, 3H), 2.81 – 2.61 (m, 4H), 2.04 (s, 3H).

(6-Cyclopropyl-3-(4-fluoro-3-methoxybenzyl)-8-((2-methyl-1H-imidazol-1-yl)methyl)-4,5-dihydro-3H-naphtho[1,2-d][1,2,3]triazole (**C3TD343**):

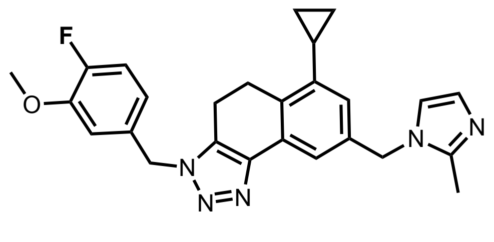

C3TD343 was prepared following the procedures described in **C16-TZ**, substituting 4-(azidomethyl)-2-methoxy-1-fluoro-benzene (1.2 equiv., 652 mg, 3.6 mmol) in Intermediate **7** reacting with common intermediate dibromide **6** (1.2 g, 3 mmol) to afford

the respective triazole intermediate **8** (1.5 g, 85% yield, 5-[2-[2,6-dibromo-4-(tetrahydropyran-2-yloxymethyl)phenyl]ethyl]-1-[(4-fluoro-3-methoxy-phenyl)methyl]triazole). Using 309 mg (0.53 mmol) of 5-[2-[2,6-dibromo-4-(tetrahydropyran-2-yloxymethyl)phenyl]ethyl]-1-[(4-fluoro-3-methoxy phenyl)methyl]triazole into Step 7 the two-step/one-pot CH activation ring closure and Suzuki reaction substituting cyclopropylboronic acid (1.1 equiv., 45 mg, 0.53 mmol) proceed smoothly. After flash chromatography purification (0-100% EtOAc/hexanes) 144 mg of the desired respective tricyclic core was isolated (58% yield). The subsequent deprotection, activation, and displacement (Step 10) steps substituting 2-methylimidazole as the S2 amine was performed on 0.7 mmol scale in the final step (4 equiv., 229 mg, 2.8 mmol). After purification using RP-HPLC the product fractions were pooled, concentrated under nitrogen stream and neutralized using NaOH. The free base product was extracted using DCM, washed with brine, filtered from Na_2SO_4, and dried under vacuum to afford 42 mg the title product as a white solid (15%): Purity ≥95% by LCMS (210, 254 nm); t_R = 1.87 min, *m/z* = 444.52 $[M+H]^+$; ^1H NMR (400 MHz, MeOD) δ 7.50 (s, 1H), 7.12 – 7.02 (m, 3H), 6.86 (s, 1H), 6.83 – 6.73 (m, 2H), 5.57 (s, 2H), 5.13 (s, 2H), 3.84 (s, 3H), 3.22 (t, *J* = 7.9 Hz, 2H), 2.90 (t, *J* = 7.9 Hz, 2H), 2.31 (s, 3H), 1.97 – 1.86 (m, 1H), 0.98 – 0.89 (m, 2H), 0.59 – 0.53 (m, 2H).

(3-(3,5-Dimethoxybenzyl)-8-((2-methyl-1*H*-imidazol-1-yl)methyl)-6-(1-methyl-3-(trifluoromethyl)-1*H*-pyrazol-4-yl)-4,5-dihydro-3*H*-naphtho[1,2-*d*][1,2,3]triazole (**C3TD078**):

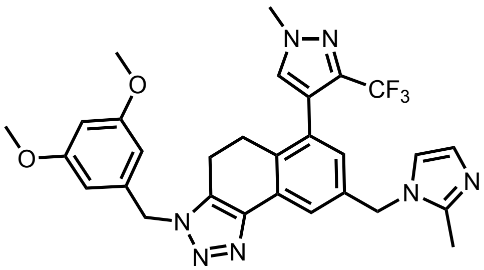

C3TD078 was prepared following the procedures described in **C16-TZ**, starting from the common tricyclic S7 containing intermediate **8** the two-step/one-pot CH activation ring closure and Suzuki reaction (Step 7) was conducted substituting [1-methyl-3-(trifluoromethyl)pyrazol-4-yl]boronic acid (2 equiv., 391 mg, 2.0 mmol). Upon consumption of starting material as determined by LCMS the acid mediated deprotection was performed (Step 8). The crude was purified by automated flash chromatography purification (0-100% EtOAc/hexanes) to afford 190 mg of the desired respective tricyclic core carbinol (38% yield, Purity ≥95% by LCMS (210, 254 nm); t_R = 1.31 min, *m/z* = 505.52 $[M+H]^+$). The subsequent activation and displacement (Steps 9-10) was performed using 2-methylimidazole as the S2 amine on 3 mmol scale in the final step (4 equiv., 1.3 g). After purification using RP-HPLC the product fractions were pooled, concentrated under nitrogen stream and neutralized using NaOH. The free base product was extracted using DCM, washed with brine, filtered from Na_2SO_4, and dried under vacuum to afford 310 mg the title product as a white solid (55%): Purity ≥95% by LCMS (210, 254 nm); t_R = 1.89 min, *m/z* = 564.23 $[M+H]^+$; ^1H NMR (400 MHz, MeOD) δ 7.71 (d, *J* = 13.2 Hz, 2H), 7.08 (s, 1H), 6.86 (s, 2H), 6.47 – 6.33 (m, 3H), 5.53 (s, 2H), 5.22 (s, 2H), 3.97 (s, 3H), 3.73 (s, 6H), 2.88 – 2.73 (m, 4H), 2.32 (s, 3H).

6-Cyclopropyl-3-[(4-methoxy-2-pyridyl)methyl]-8-[(2-methylimidazol-1-yl)methyl]-4,5-dihydrobenzo[e]benzotriazole (**C3TD424**):

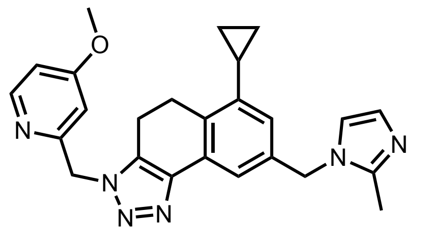

C3TD424 was prepared following the procedures described in **C16-TZ**. Intermediate **6** was reacted in sequence with Steps 6-9, substituting 2-(azidomethyl)-4-methoxy-pyridine (1 equiv., 408.24 mg, 2.49 mmol) in Step 6 and substituting cyclopropylboronic acid (1.1 equiv., 136.16 mg, 1.59 mmol) in Step 7, and substituting 2-methylimidazole (2 equiv., 38.02 mg, 0.463 mmol) in the displacement Step 10, with addition of free basing the product TFA salt with aq. NaOH after RP-HPLC purification. Upon solvent removal under reduced pressure and further drying in vacuo the title product was obtained as a white solid (18 mg, 0.042 mmol, 18%): Purity >95% by LCMS (215, 254 nm); t_R = 1.431 min, *m/z* = 427.2251 $[M+H]^+$; ^1H NMR (400 MHz, CDCl3) δ 8.39 (d, J = 5.8 Hz, 1H), 7.70 (s, 1H), 7.01 (d, J = 2.1 Hz, 1H), 6.82 (s, 1H), 6.78 (s, 1H), 6.71 (s, 1H), 5.68 (s, 2H), 5.15 (d, J = 27.1 Hz, 2H), 3.84 (s, 3H), 3.25 (q, J = 7.9 Hz, 2H), 3.01 (t, J = 8.1 Hz, 2H), 2.80 (s, 3H), 1.25 (s, 1H), 1.03 – 0.93 (m, 2H), 0.56 (d, J = 5.4 Hz, 2H). ^{13}C NMR (101 MHz, CDCl3) δ 166.76, 156.71, 150.88, 150.79, 140.35, 137.21, 136.58, 132.90, 131.34, 124.94, 109.35, 109.02, 108.01, 107.49, 98.07, 66.87, 62.24, 55.27, 53.26, 35.52, 30.42, 25.35, 21.51, 19.25.

SUPPLEMENTAL FIGURES

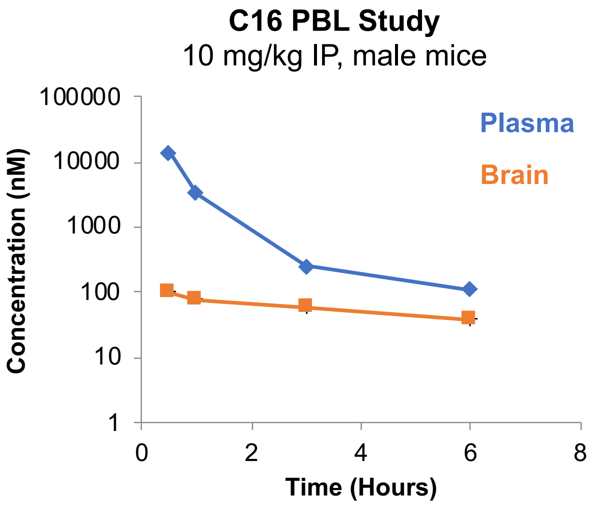

	Plasma	Brain
AUC (nM*hrs)	11,900	357
AUC Interval (hrs)	6	6
C_{max} (nM)	14,200	102
T_{max} (hrs)	0.5	0.5
**P:B Ratio (AUC)**	0.03	

Supplemental Figure 1. Snapshot plasma:blood level (PBL) study for **C16** dosed at 10 mg/kg IP in male mice. **C16** was formulated in 20% beta-cyclodextrin at 2 mg/mL. Raw concentration time-course (left, mean values from n = 2 mice) and extracted values (right) are shown.

PDB ID	9NCW	9NCV	9NCT
Beamline	APS LS-CAT 21-ID-F	APS LS-CAT 21-ID-F	APS LS-CAT 21-ID-F
Wavelength (A)	0.97872	0.97872	0.97872
Resolution range	38.23 - 1.58 (1.6 - 1.58)	45.5 - 1.583 (1.6 - 1.58)	44.67 - 2.106 (2.16 - 2.11)
Space group	P 1	P 1	P 1 21 1
Unit cell	46.53 68.69 93.01 91.29 103.26 90.38	46.76 68.83 94.38 89.40 76.65 89.97	69.00 46.88 93.328 90.00 113.52 90.00
Total reflections	558098	538443	204477
Unique reflections	144679 (3473)	148776 (4019)	30647 (1951)
Multiplicity	3.8 (3.3)	3.6 (2.9)	6.7 (6.0)
Completeness (%)	94.95 (67.20)	95.28 (76.15)	95.45 (86.25)
Mean I/sigma(I)	11.03 (1.06)	14.52 (1.76)	19.96 (3.26)
Wilson B-factor	14.53	14.22	25.78
R-merge	0.101 (0.577)	0.079 (0.305)	0.117 (0.526)
R-meas	0.125 (0.690)	0.101 (0.438)	0.138 (0.552)
R-pim	0.064 (0.372)	0.053 (0.246)	0.053 (0.221)
CC1/2	0.981 (0.836)	0.992 (0.923)	0.993 (0.862)
CC*	0.995 (0.954)	0.998 (0.980)	0.998 (0.962)
Reflections used in refinement	144679 (3473)	148776 (4019)	30647 (1951)
Reflections used for R-free	7045 (182)	7593 (168)	1993 (125)
R-work	0.2491 (0.4321)	0.2528 (0.2870)	0.1754 (0.2088)
R-free	0.2753 (0.4238)	0.2873 (0.3155)	0.2282 (0.2751)
Number of non-hydrogen atoms	10305	10456	4873
macromolecules	9074	9067	4539
ligands	80	160	68
solvent	1151	1229	266
Protein residues	1184	1184	592
RMS(bonds)	0.006	0.055	0.006
RMS(angles)	0.83	2.67	0.86
Ramachandran favored (%)	96.23	97.00	95.72
Ramachandran allowed (%)	3.77	3.00	4.28
Ramachandran outliers (%)	0	0	0
Rotamer outliers (%)	0.1	0.2	0.4
Clashscore	2.73	2.44	3.99
Average B-factor	16.97	16.08	27.01
macromolecules	16	15.09	26.8
ligands	19.44	16.61	29.06
solvent	24.43	23.34	30.06

Supplemental Figure 2. Table 1 data collection and refinement statistics for the three WDR5 co-crystal structures reported herein. One crystal was used for each structure. Values in parentheses unless stated represent data in the highest-resolution shell. RMS = root mean square.

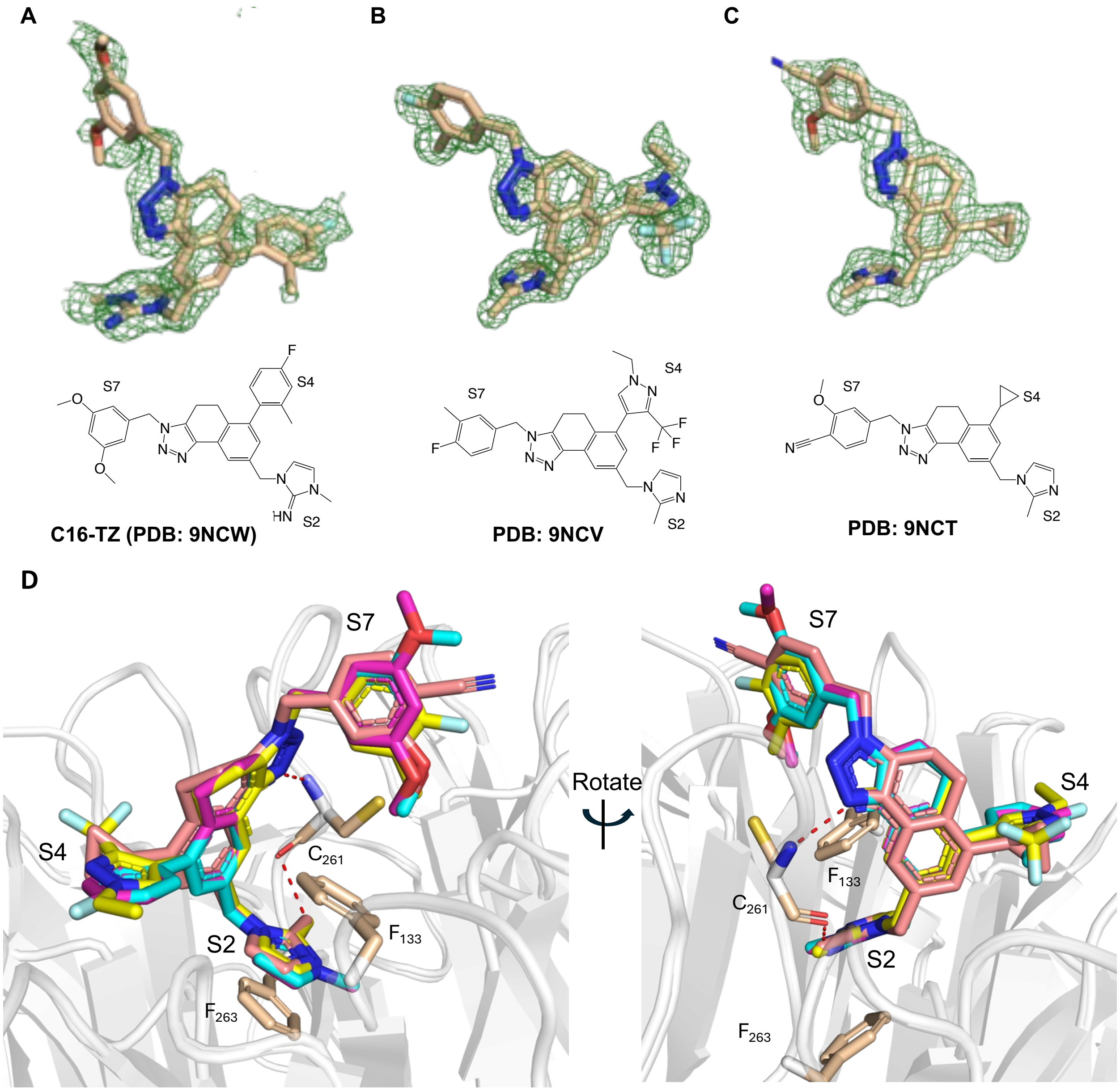

Supplemental Figure 3. Additional x-ray crystal structures of WDR5 in complex with triazole based inhibitors with differing S2, S4, and S7 pieces. Ligand Polder OMIT maps contoured to σ = 3.0 and shown for Chain A ligands **(A) C16-TZ**, PDB: 9NCW, **(B)** PDB: 9NCV and (**C)** PDB: 9NCT. **(D)** Alignment of ligands highlights a conserved binding pose (**C16-TZ** – blue sticks, 9NCV – yellow sticks, 9NCT– pink sticks).

Test Article	Direction	Recovery (%)	P_{app} (10-6 cm/s)			Efflux Ratio	Brain Penetration Classification
			R1	R2	AVG		
**C16**	A-to-B	85.6	0.556	0.875	0.715	2.38	Low
	B-to-A	87.3	1.49	1.91	1.70		
**CCF343**	A-to-B	71.2	0.131	0.0949	0.113	383	Low
	B-to-A	76.8	40.2	46.3	43.3		

Supplemental Figure 4. Blood-brain barrier (BBB) penetration potential using MDR1-MDCK cell monolayers. All P-glycoprotein (MDR1) permeability and efflux studies were conducted at Absorption Systems (parent company Pharmaron). In brief, MDR1-MDCK monolayers were grown to confluence on collagen-coated, microporous membranes in 12-well assay plates. The permeability assay buffer was Hanks' balanced salt solution containing 10 mM HEPES and 15 mM glucose at a pH of 7.4. The buffer in the receiver chamber also contained 1% bovine serum albumin. The dosing solution concentration was 5 µM of test article in the assay buffer. Cell monolayers were dosed on the apical side (A-to-B) or basolateral side (B-to-A) and incubated at 37°C with 5% CO2 in a humidified incubator. Samples were taken from the donor and receiver chambers after 120 minutes. Each determination was performed in duplicate. The flux of lucifer yellow was also measured post-experimentally for each monolayer to ensure no damage was inflicted to the cell monolayers during the flux period. All samples were assayed by LC-MS/MS using electrospray ionization. Efflux ratio (ER) is defined as Papp (B-to-A) / Papp (A-to-B).

Brain Penetration Potential Classification:
P_{app} (A-to-B) ≥ 3.0 (10^{-6} cm/s) and ER < 3.0: **High**
P_{app} (A-to-B) ≥ 3.0 (10^{-6} cm/s) and 10 > ER ≥ 3.0: **Moderate**
P_{app} (A-to-B) ≥ 3.0 (10^{-6} cm/s) and ER ≥ 10, or Papp (A-to-B) < 3.0 (10^{-6} cm/s): **Low**

A

L0 CSCs, 200 nM C3TD078 vs. DMSO

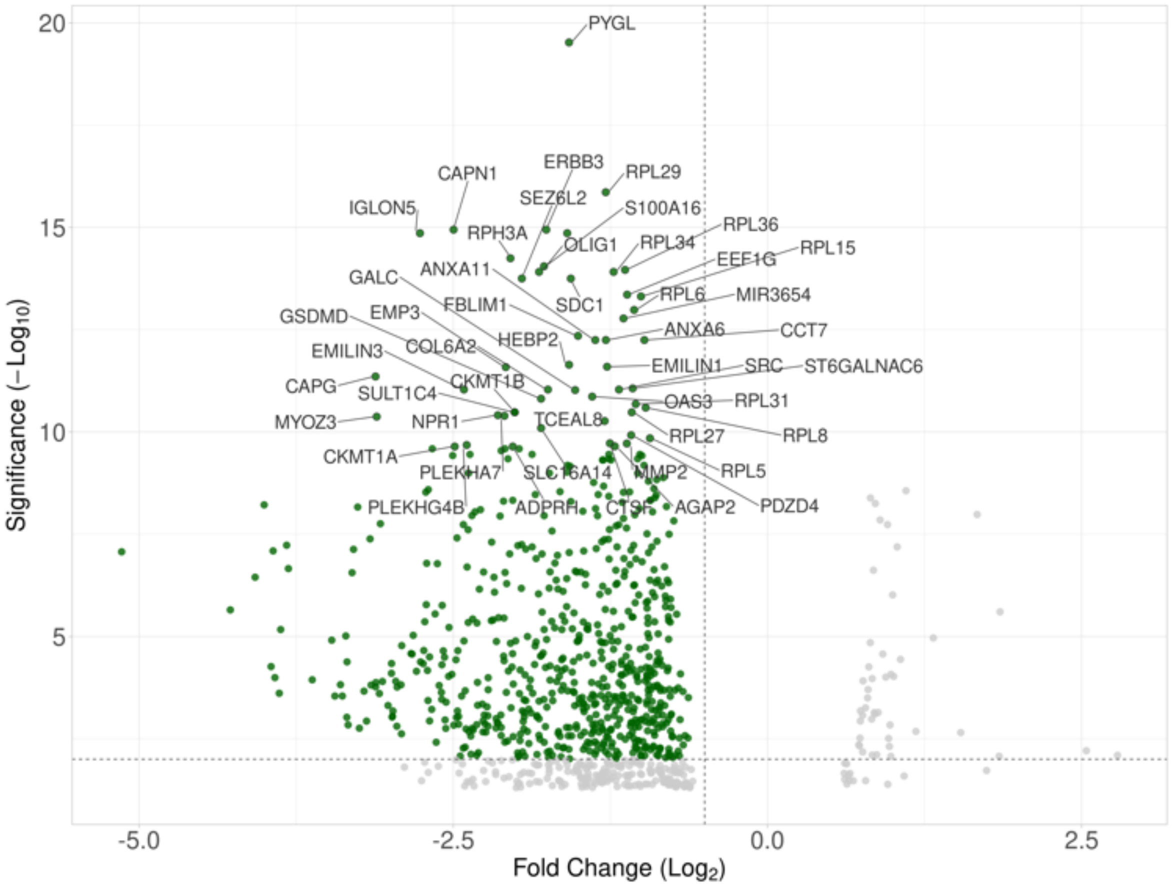

GO Biological Process Enrichment

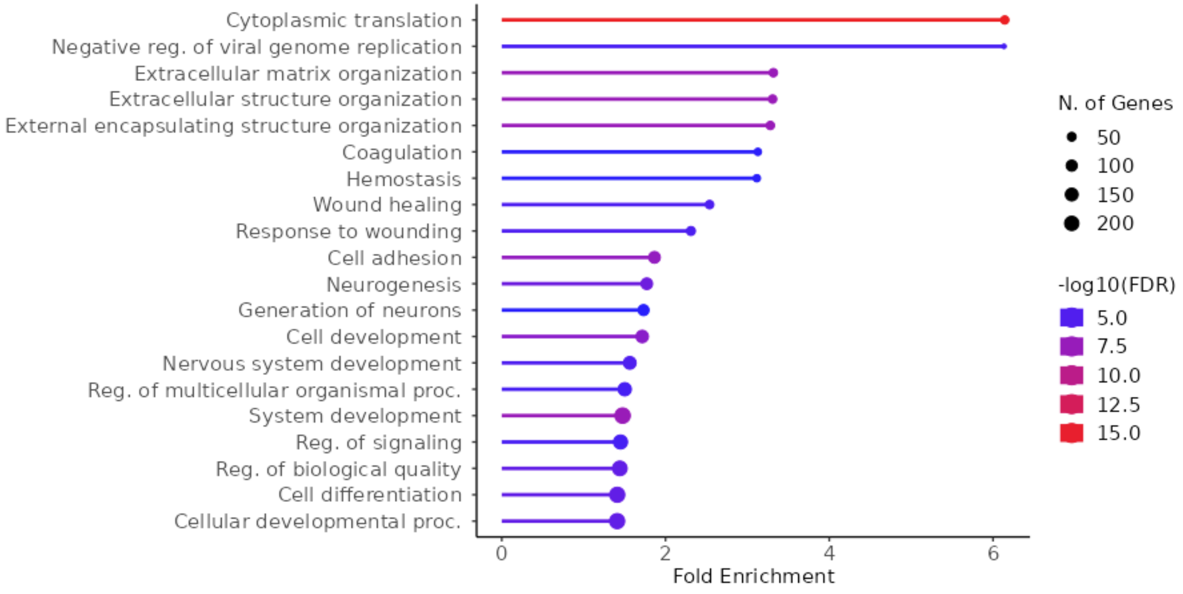

B

DI318 CSCs, 200 nM C3TD078 vs. DMSO

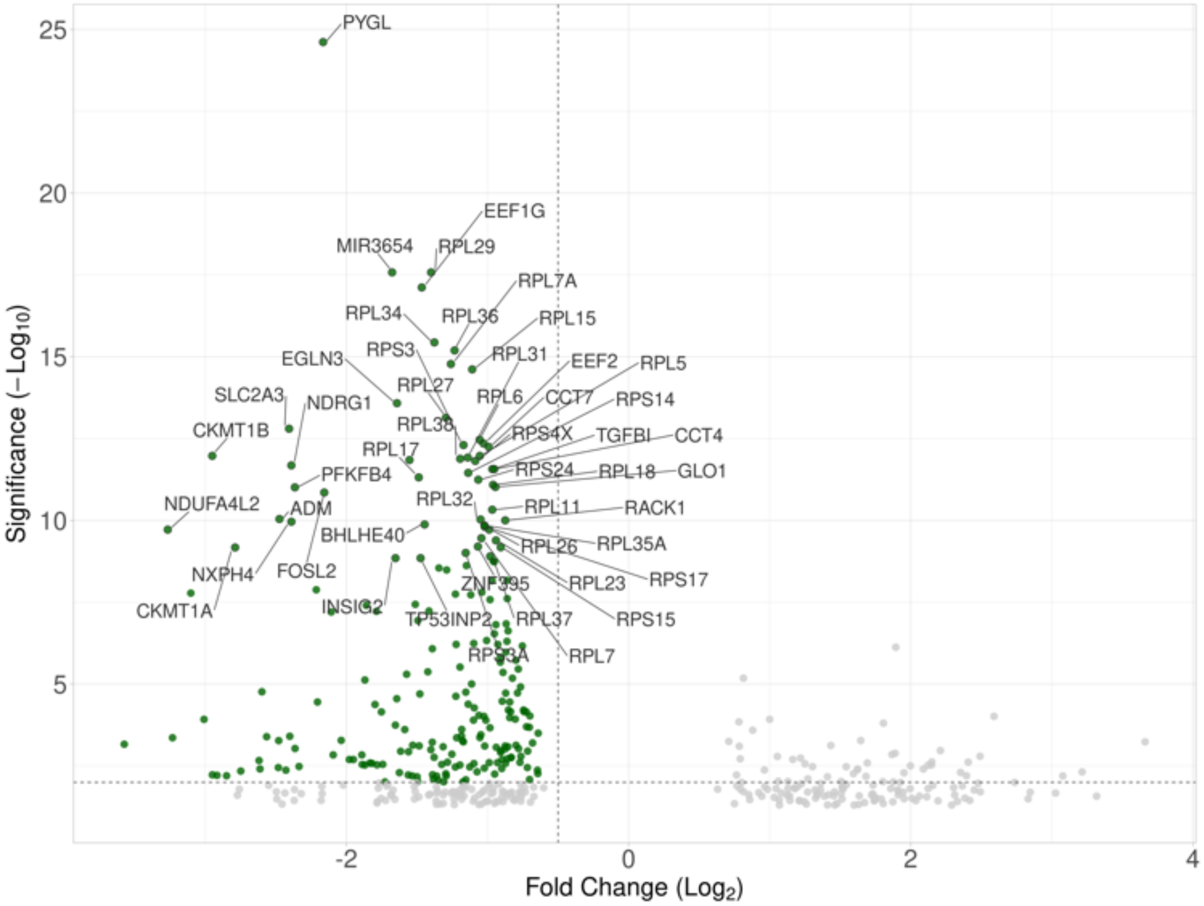

GO Biological Process Enrichment

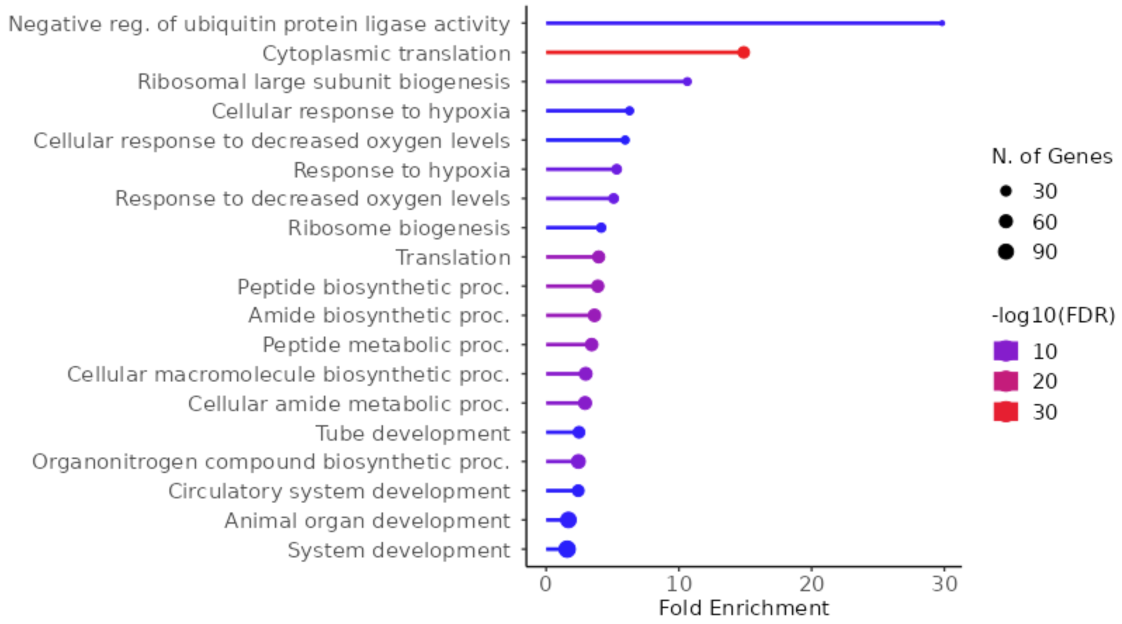

C
L0 CSCs, 200 nM C16 vs. DMSO

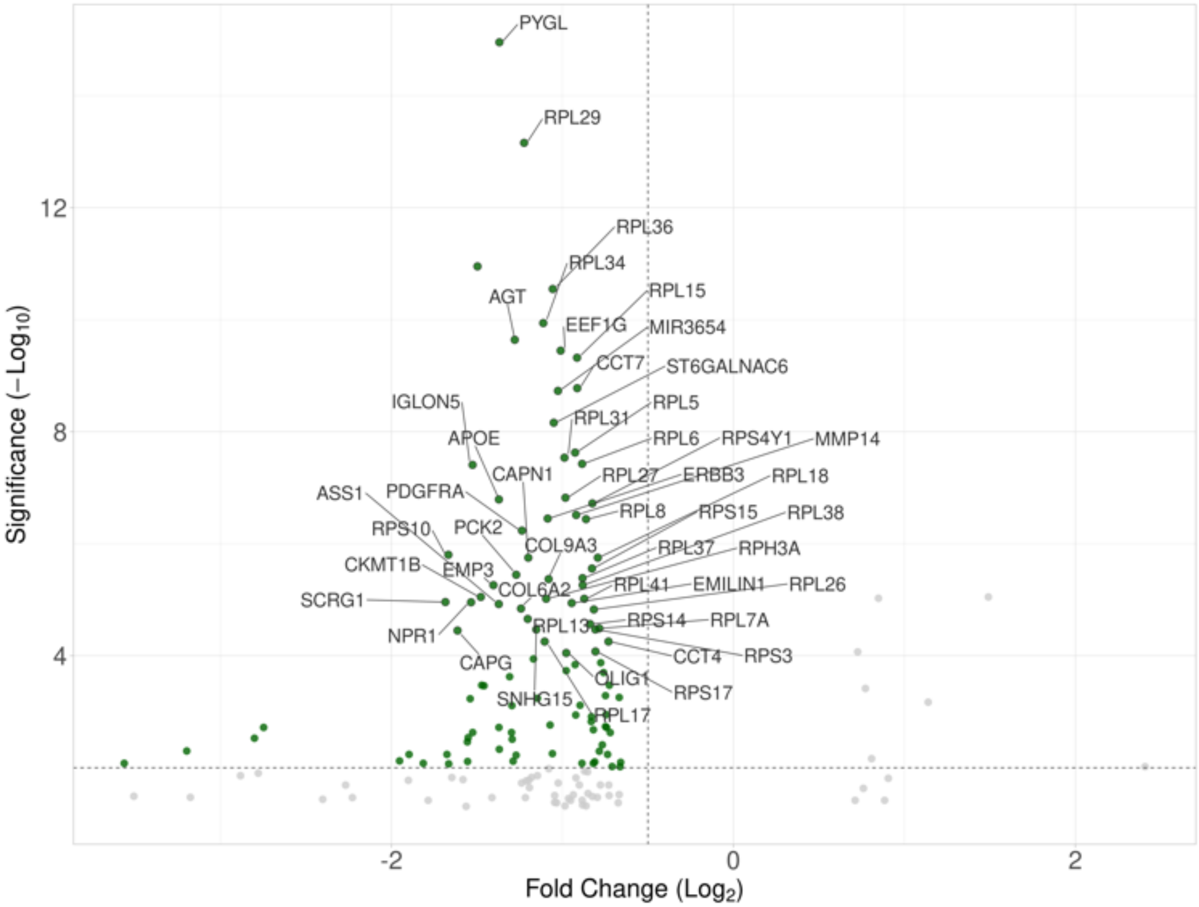

GO Biological Process Enrichment

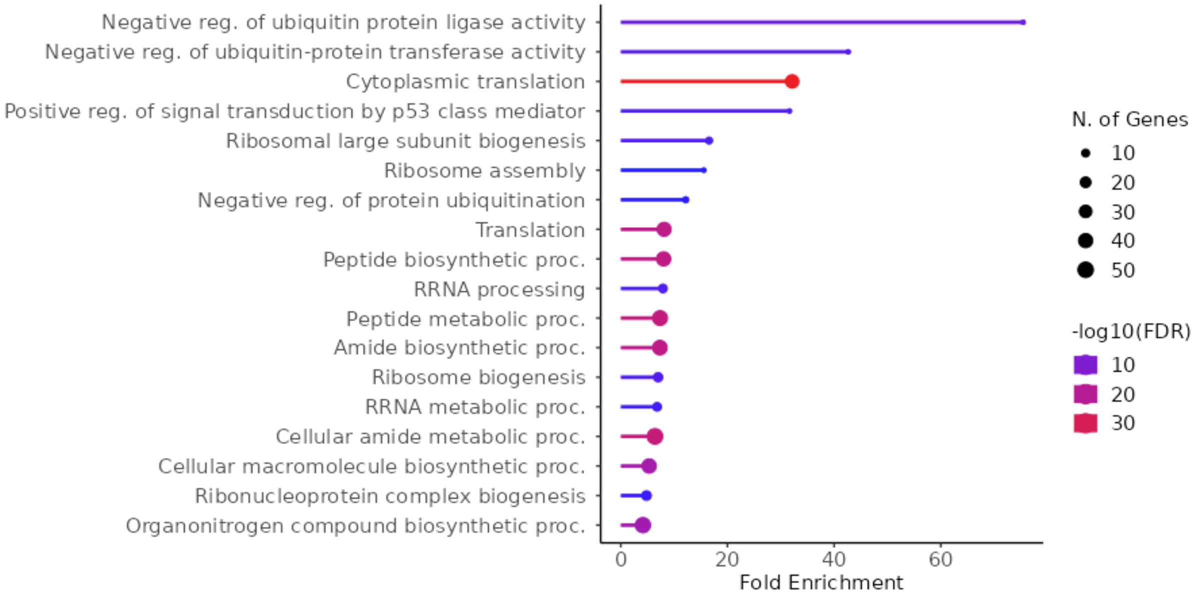

D

DI318 CSCs, 200 nM C3TD078 vs. DMSO

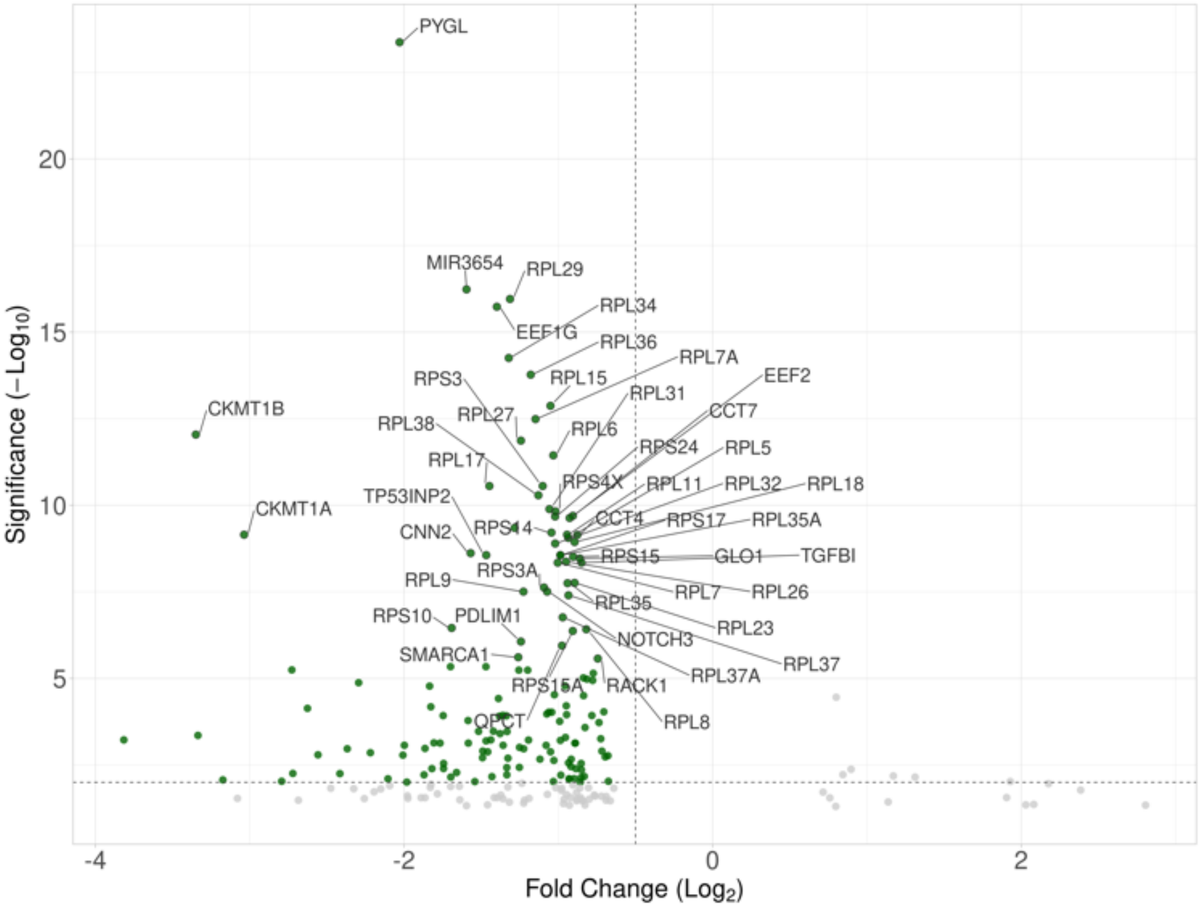

GO Biological Process Enrichment

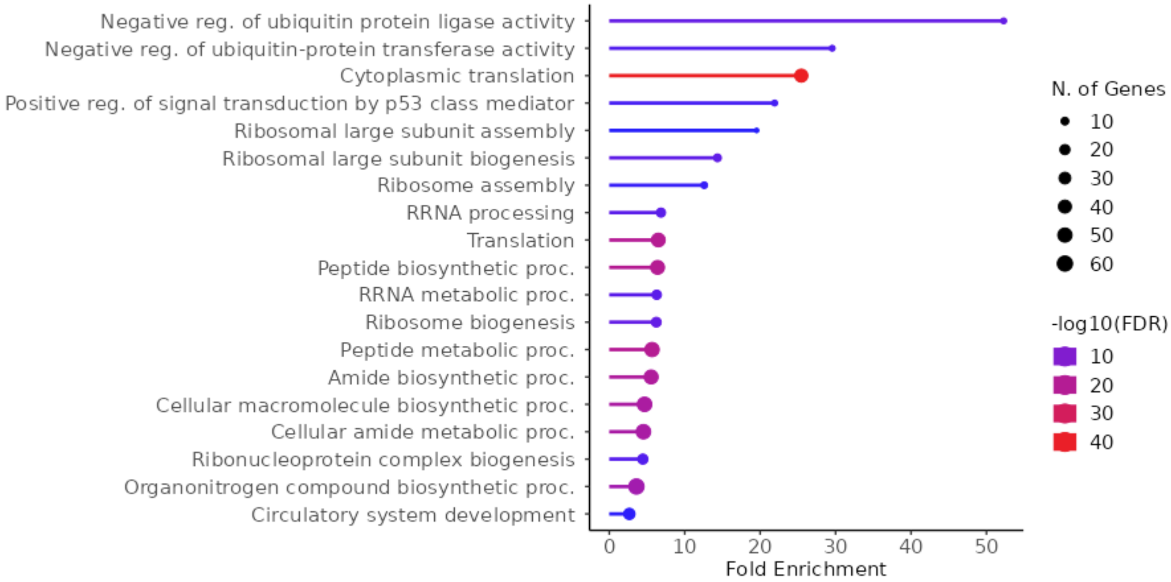

Supplemental Figure 5. Volcano plots (differentially downregulated genes in green, top 50 annotated) and biological processes GO enrichment for **C3TD879** in **(A)** L0 and **(B)** DI318 CSCs**,** as well as **C16** in **(C)** L0 and **(D)** DI318 CSCs.

- PYGL
- RPL29
- SDC1
- RPL34
- RPL36
- EEF1G
- RPL15
- RPL6
- EMILIN3
- CCT7
- CKMT1B
- CKMT1A
- RPL31
- RPL8
- RPL27
- RPL5
- RPS10
- RPL41
- RPL38
- ELF4
- RPS17
- RPS15
- RPL9
- RPL26
- RPS24
- RPS14
- RPL37
- CCT4
- RPL17
- GLO1
- RPL32
- RPS4X
- RPS3A
- RPL7
- RPL23
- RPL7A
- RPS18
- RPL35A
- RPL18
- RPL35
- RPS3
- RPS15A
- RPL11

Supplemental Figure 6. Core set of WIN-site regulated genes in CSCs. These genes were downregulated ($p < 0.01$; $log_2FC < -0.5$) by both **C3TD078** and **C16** in both L0 and DI318 CSC models as measured by bulk RNAseq.

A

GO TERM	L0 CSCs (FDR)		DI318 CSCs (FDR)	
	C16	CCF078	C16	CCF078
GO:0002181 **Cytoplasmic translation**	7.4×10^{-40}	2.6×10^{-16}	1.6×10^{-41}	1.5×10^{-32}
GO:0042254 **Ribosome biogenesis**	3.2×10^{-6}	*n.s.*	2.7×10^{-7}	1.4×10^{-7}
GO:0022008 **Neurogenesis**	*n.s.*	1.7×10^{-6}	*n.s.*	*n.s.*
GO:0001666 **Response to hypoxia**	*n.s.*	*n.s.*	*n.s.*	1.7×10^{-8}

B

Reactome Pathway	Entities Found	Entities Total	FDR
**Translation**	40	294	$< 3.3 \times 10^{-16}$
**rRNA Processing**	39	203	$< 3.3 \times 10^{-16}$
**Peptide Chan Elongation**	39	90	$< 3.3 \times 10^{-16}$
**rRNA Processing in the Nucleus and Cytosol**	39	193	$< 3.3 \times 10^{-16}$

Supplemental Figure 7. (A) Highlighted enriched GO terms (biologically processes) across cell lines and treatments. **(B)** Pathway enrichment analysis using Reactome for the "core" set of WIN-site dependent CSC genes highlighted in **Supplemental Figure 6**.

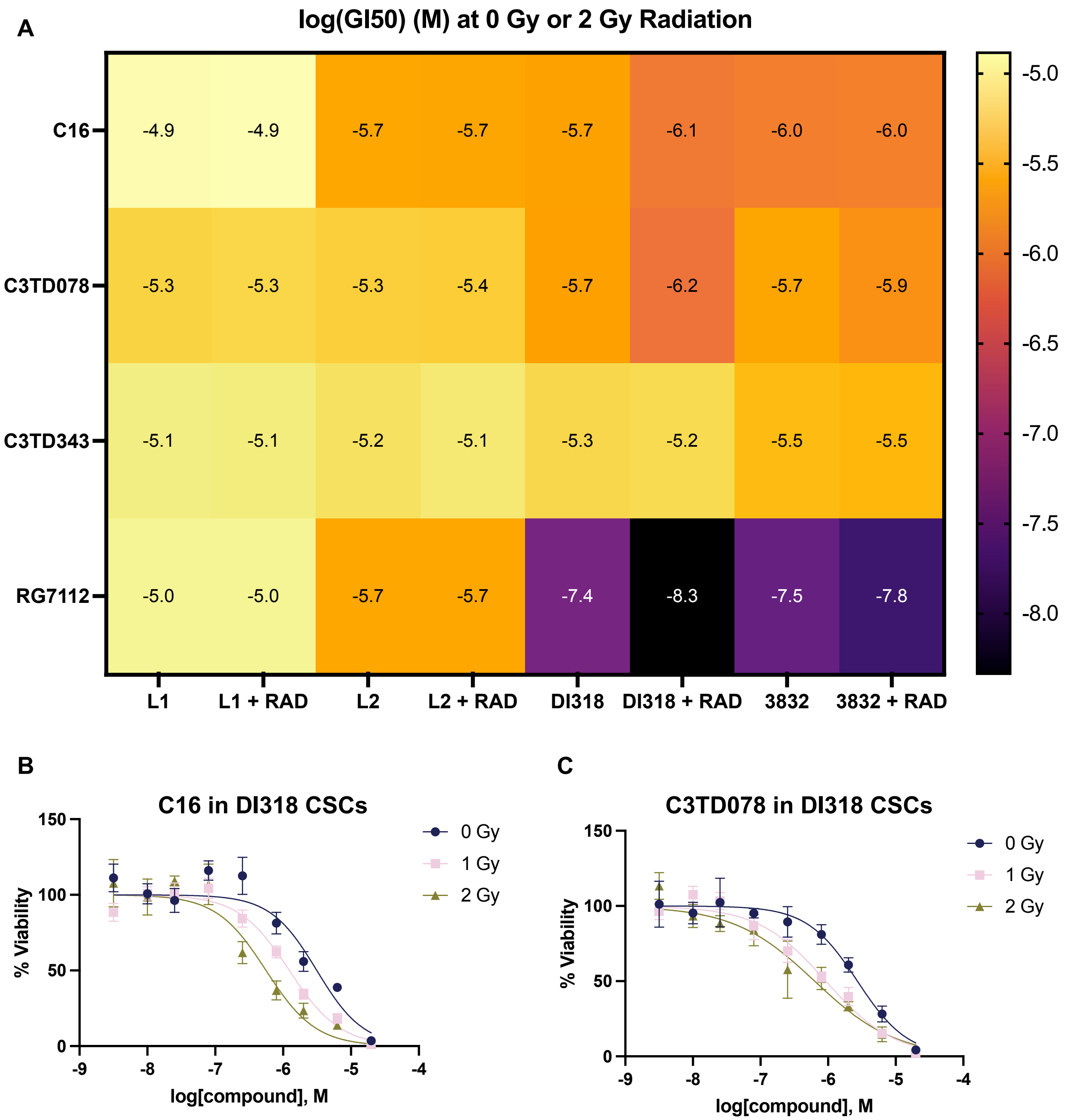

Supplemental Figure 8. **(A)** pGI50 (-log[GI50]] (M) values for L1, L2, DI318, and 3832 CSC models treated with a dose-response of **C16**, **C3TD078, C3TD343**, or the MDM2 antagonist (p53 activator) **RG7112** following either 0 Gy or 2 Gy irradiation. Mean values are presented from a single biological replicate conducted in technical triplicate. Follow-up experiments in just DI318 CSCs pre-treated for three days with either **(B) C16** and **(C) C3TD078** following irradiation with either 0, 1, or 2 Gy. Cell viability was quantified as described in the Materials and Methods four days post-irradiation (seven days total). The sensitivity of DI318 CSCs to WIN-site inhibitors was modestly enhanced by radiation.

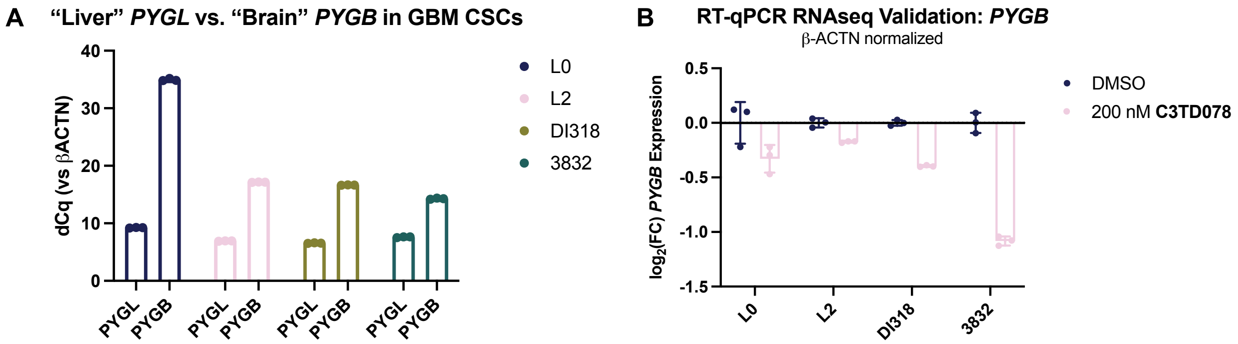

Supplemental Figure 9. **(A)** Expression (dCq vs ACTB) for the liver *PYGL* isoform and the brain *PYGB* isoform in L0, L2, DI318, and 3832 CSCs as quantified by RT-qPCR. The “liver” *PYGL* is expressed at a much higher level than the “brain” *PYGB*. **(B)** Unlike *PYGL,* the expression of *PYGB* is largely unaffected ($log_2FC < 1.0$) in L0, L2, DI318, and 3832 cells treated for 72 hours with 200 nM **C3TD078.**

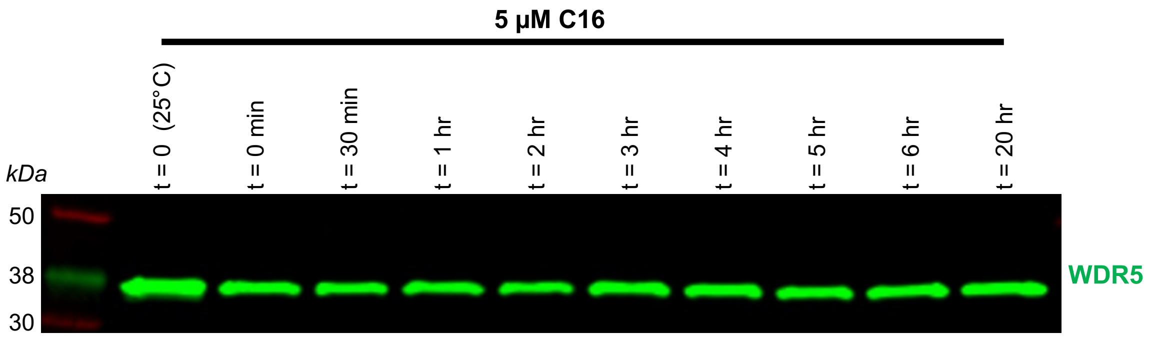

Supplemental Figure 10. L0 CSCs were treated for 2 hours with 5 µM **C16** prior to compound washout, and samples were prepared for CETSA WB at the indicated time points post-compound removal. The first sample was not heated and represents total cellular WDR5, while the other lanes were heated to 70°C to remove unbound WDR5 (see Materials and Methods). Like **C3TD078**, **C16** remains bound to WDR5 in L0 CSCs for at least 20 hours following washout.

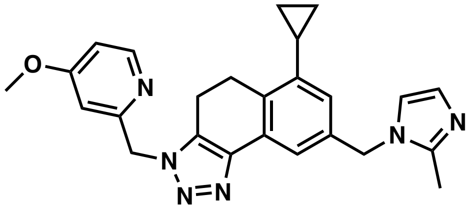

	C3TD424
MW (g/mol)<br>CLogP<br>TPSA (Å^2)	426.52<br>2.3<br>65.2
TR-FRET K_i (nM)	1.6 +/- .07 (n = 3)
CETSA K_d (nM)	500<br>(n = 1)

Supplemental Figure 11. Chemical structure and *in vitro* profile of **C3TD424**, the weakly potent inhibitor related to **C3TD343**, that was used to validate the on-target nature of the transcriptional responses presented in **Figure 3**.

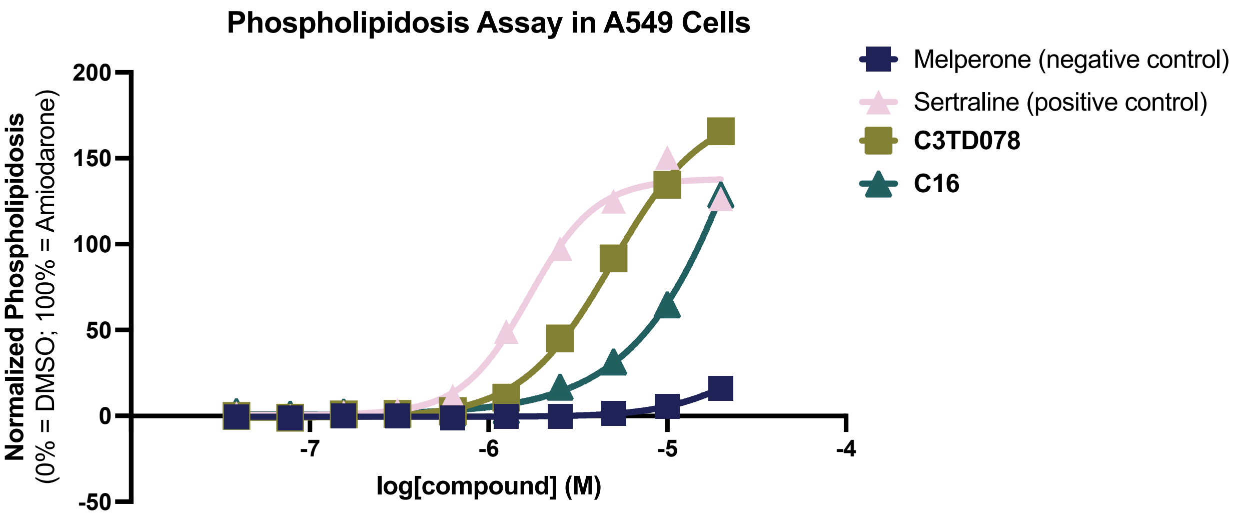

Supplemental Figure 12. The ability of **C3TD078** and **C16** to induce phospholipidosis (PLD) was measured using the immunofluorescence assay reported by Tummino *et al.* 2021 (2). Briefly, A549 cells were treated for 24 hours with compounds in the presence of the lipid dye NBD-PE (ThermoFisher #N360), which fluoresces green as it accumulates in vesicular lipid bodies. Total green intensity was measured using a Cytation 5 plate reader and normalized to DMSO-treated (0%) and amiodarone-treated (100%) cells. The positive control sertraline and both **C16**/**C3TD078** induced PLD in A549 cells at doses > 1 µM, with maximum levels at 20 µM compound exceeding 100% of the level induced by amiodarone. A negative control compound, melperone, was unable to induce PLD as expected. Each data point represents a single technical replicate (one well, all data shown in figure).

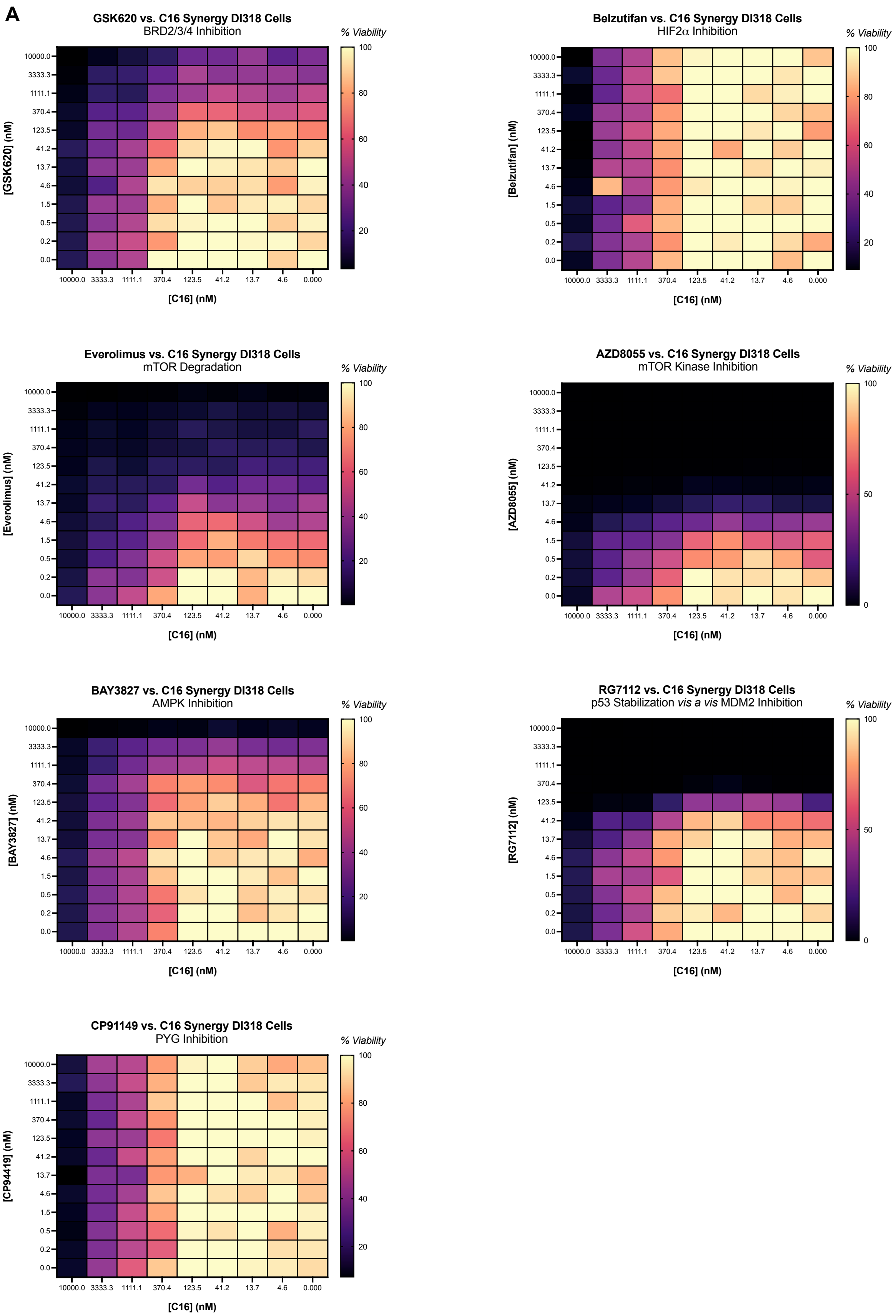
A
GSK620 vs. C16 Synergy DI318 Cells
BRD2/3/4 Inhibition
% Viability
[GSK620] (nM)
[C16] (nM)
Belzutifan vs. C16 Synergy DI318 Cells
HIF2α Inhibition
% Viability
[Belzutifan] (nM)
[C16] (nM)
Everolimus vs. C16 Synergy DI318 Cells
mTOR Degradation
% Viability
[Everolimus] (nM)
[C16] (nM)
AZD8055 vs. C16 Synergy DI318 Cells
mTOR Kinase Inhibition
% Viability
[AZD8055] (nM)
[C16] (nM)
BAY3827 vs. C16 Synergy DI318 Cells
AMPK Inhibition
% Viability
[BAY3827] (nM)
[C16] (nM)
RG7112 vs. C16 Synergy DI318 Cells
p53 Stabilization vis a vis MDM2 Inhibition
% Viability
[RG7112] (nM)
[C16] (nM)
CP91149 vs. C16 Synergy DI318 Cells
PYG Inhibition
% Viability
[CP94419] (nM)
[C16] (nM)
10000.0
3333.3
1111.1
370.4
123.5
41.2
13.7
4.6
1.5
0.5
0.2
0.0
0.000

B

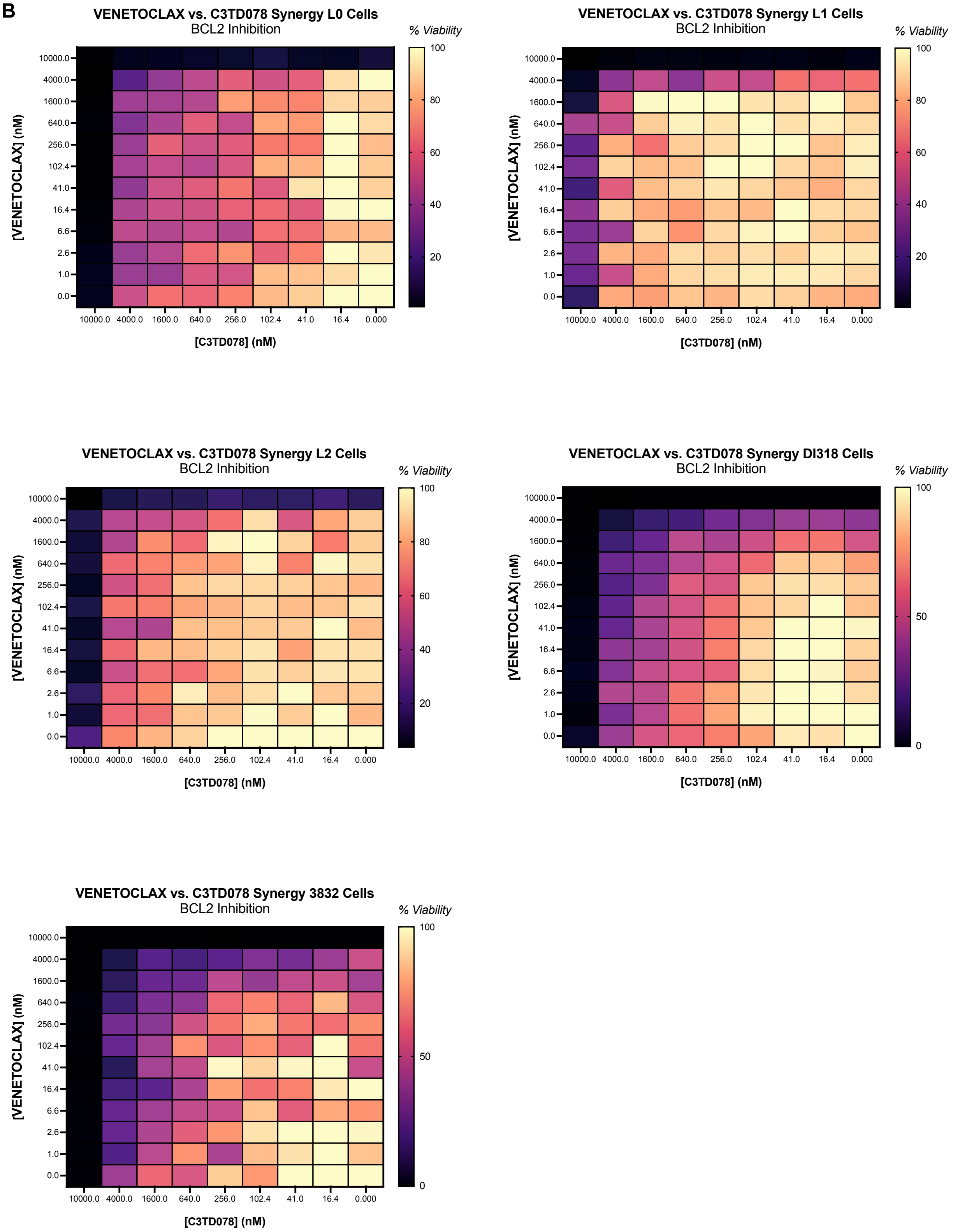

Supplemental Figure 13. (A) Cross-titration experiments with **C16** and a variety of rationally selected small-molecules in DI318 CSCs. **(B)** Cross-titration experiments with

C3TD078 and the BCL2 inhibitor Venetoclax in multiple CSC models. No drug-drug synergy was observed in any combination experiment as estimated by MuSyC (see Materials and Methods). Data are presented as % viability from a single biological replicate (one well per concentration combination, all data presented in the figure) after a 7-day treatment period.

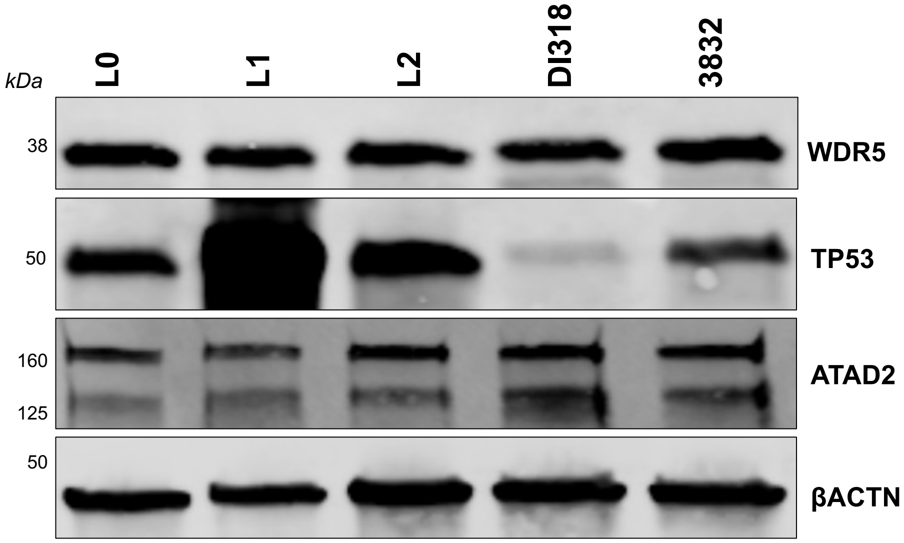

WDR5: 1:1000 rb CST1310
TP53: 1:500 ms sc-126
ATAD2: 1:800 rb CST 50563
ACTN: 1:3000 ms CST 3700

Supplemental Figure 14. Protein expression of WDR5, ATAD2, and p53 in L0, L1, L2, DI318, and 3832 CSCs as measured by western blot with the indicated antibodies.

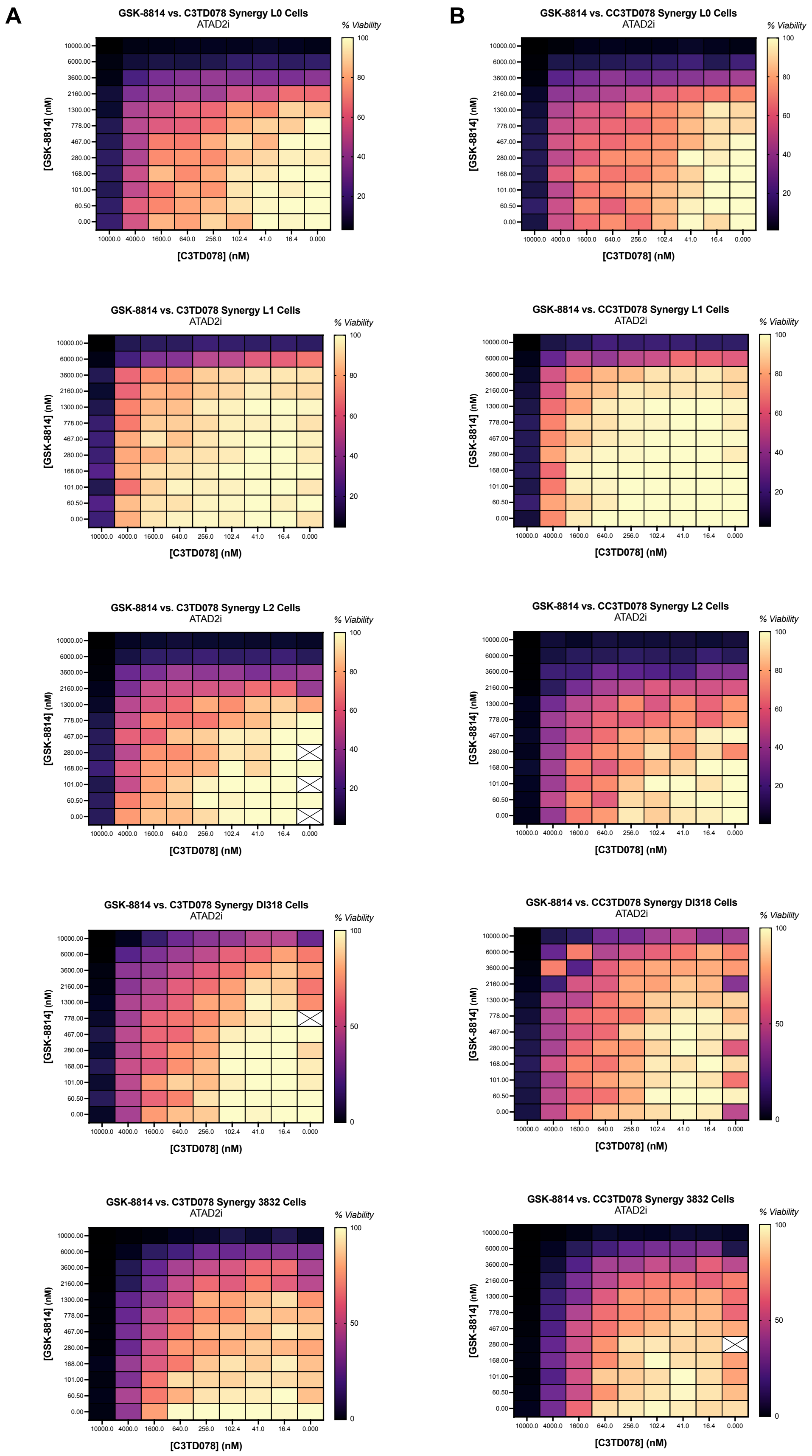
A
GSK-8814 vs. C3TD078 Synergy L0 Cells
ATAD2i
GSK-8814 vs. C3TD078 Synergy L1 Cells
ATAD2i
GSK-8814 vs. C3TD078 Synergy L2 Cells
ATAD2i
GSK-8814 vs. C3TD078 Synergy DI318 Cells
ATAD2i
GSK-8814 vs. C3TD078 Synergy 3832 Cells
ATAD2i
B
GSK-8814 vs. CC3TD078 Synergy L0 Cells
ATAD2i
GSK-8814 vs. CC3TD078 Synergy L1 Cells
ATAD2i
GSK-8814 vs. CC3TD078 Synergy L2 Cells
ATAD2i
GSK-8814 vs. CC3TD078 Synergy DI318 Cells
ATAD2i
GSK-8814 vs. CC3TD078 Synergy 3832 Cells
ATAD2i
% Viability
[GSK-8814] (nM)
10000.00
6000.00
3600.00
2160.00
1300.00
778.00
467.00
280.00
168.00
101.00
60.50
0.00
[C3TD078] (nM)
10000.0
4000.0
1600.0
640.0
256.0
102.4
41.0
16.4
0.000
100
80
60
50
40
20
0

Supplemental Figure 15. Cross-titration experiments with **C3TD078** and the ATAD2 bromodomain inhibitor GSK-8814 in five different CSC models. Data are presented as % viability from two separate biological replicates, **(A)** and **(B)**, performed as a single technical replicate. No synergy was observed as quantified by MuSyC. Points with an “X” are outliers that have been excluded from the figure for clarity.

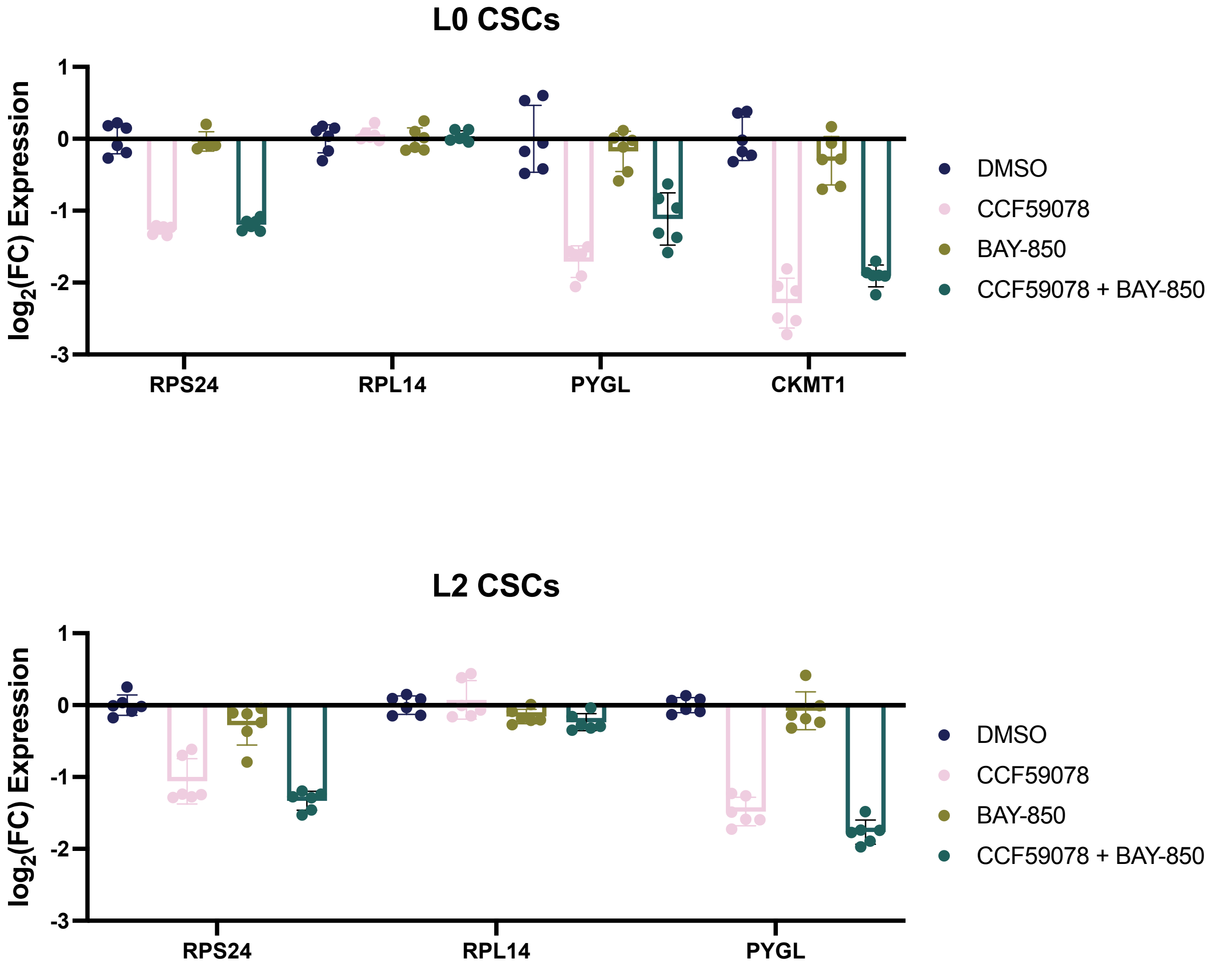

Supplemental Figure 16. CSCs were treated for 72 hours with 200 nM **C3TD078**, 700 nM **BAY-850**, or the combination and the expression of WDR5-indedpendent *RPL14* and WDR5-target genes *PYGL*, *RPS24*, and *CKMT1* were quantified by RT-qPCR. Data are representative of a single biological replicate performed in technical triplicate for each CSC model.

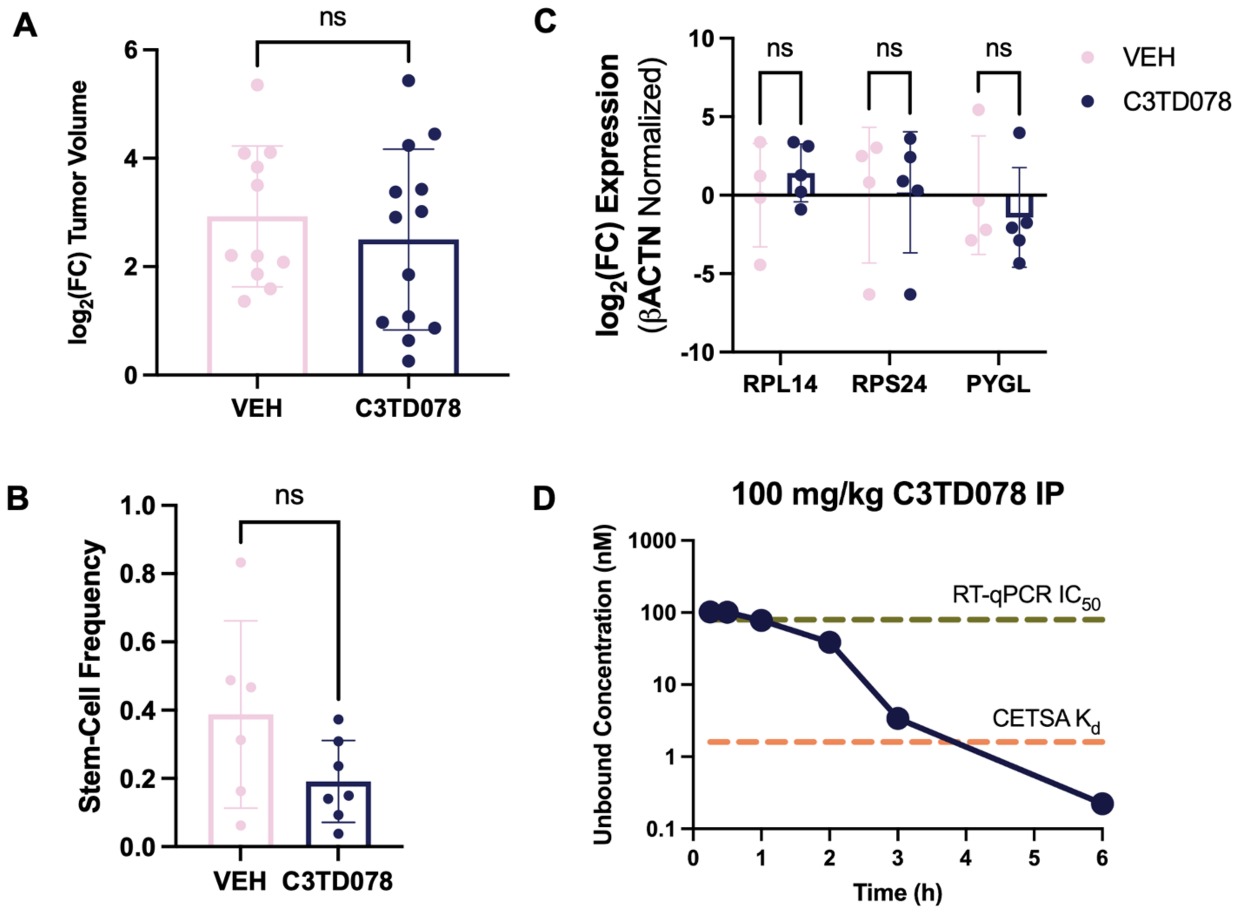

Supplemental Figure 17. (A) NOD-scid (NSG) mice were inoculated subcutaneously in the right flank with 2 × 10^6 DI318 CSCs suspended in 100 µL of PBS:Matrigel (1:1). Over the next 6-7 days tumors developed to the point where a small mass was visual. A digital caliper measurement (length and width) were taken to get a baseline tumor size. Mice were randomized into treatment groups (vehicle or **C3TD078**), ensuring even distribution of biological sex across groups. Compounds were formulated in 20% HP β-cyclodextrin (BCD) and administered once daily via IP injection at 30 mg/kg for 7 consecutive days. Endpoint ellipsoid tumor volume was calculated and compared using a t-test. **(B)** Vehicle and **C3TD078**-treated tumors were dissociated, and live cells were plated for ELDA to determine stem cell frequency as compared using a t-test. **(C)** Total unbound exposure of **C3TD078** in mice after a single injection as compared to the measured *in vitro* CETSA K_d and RT-qPCR IC_{50}. Even at this higher dose, **C3TD078** is exposed above the functional RT-qPCR IC_{50} for no more than 1 hour. Underlying data reproduced from Figure 2C. **(D)** RT-qPCR to assess downregulation of WDR5-target genes in frozen tumor samples (comparisons are t-tests).

SUPPLEMENTAL REFERENCES

1. Tian J, et al. Discovery and Structure-Based Optimization of Potent and Selective WD Repeat Domain 5 (WDR5) Inhibitors Containing a Dihydroisoquinolinone Bicyclic Core. *J Med Chem.* 2020;63(2):656-75.
2. Tummino TA, et al. Drug-induced phospholipidosis confounds drug repurposing for SARS-CoV-2. *Science.* 2021;373(6554):541-7.
