# Supplementary material for: Development and characterization of triazole-based WDR5 inhibitors for the treatment of glioblastoma
Source: JCI Insight. 2026 May 5;11(12):e198298. doi: 10.1172/jci.insight.198298 (PMC13313556; doi:10.1172/jci.insight.198298)

Corresponding to Figure 4A. Green = WDR5, red = ACTN.

Bands; used in main text figure; top = C16, bottom = DMSO

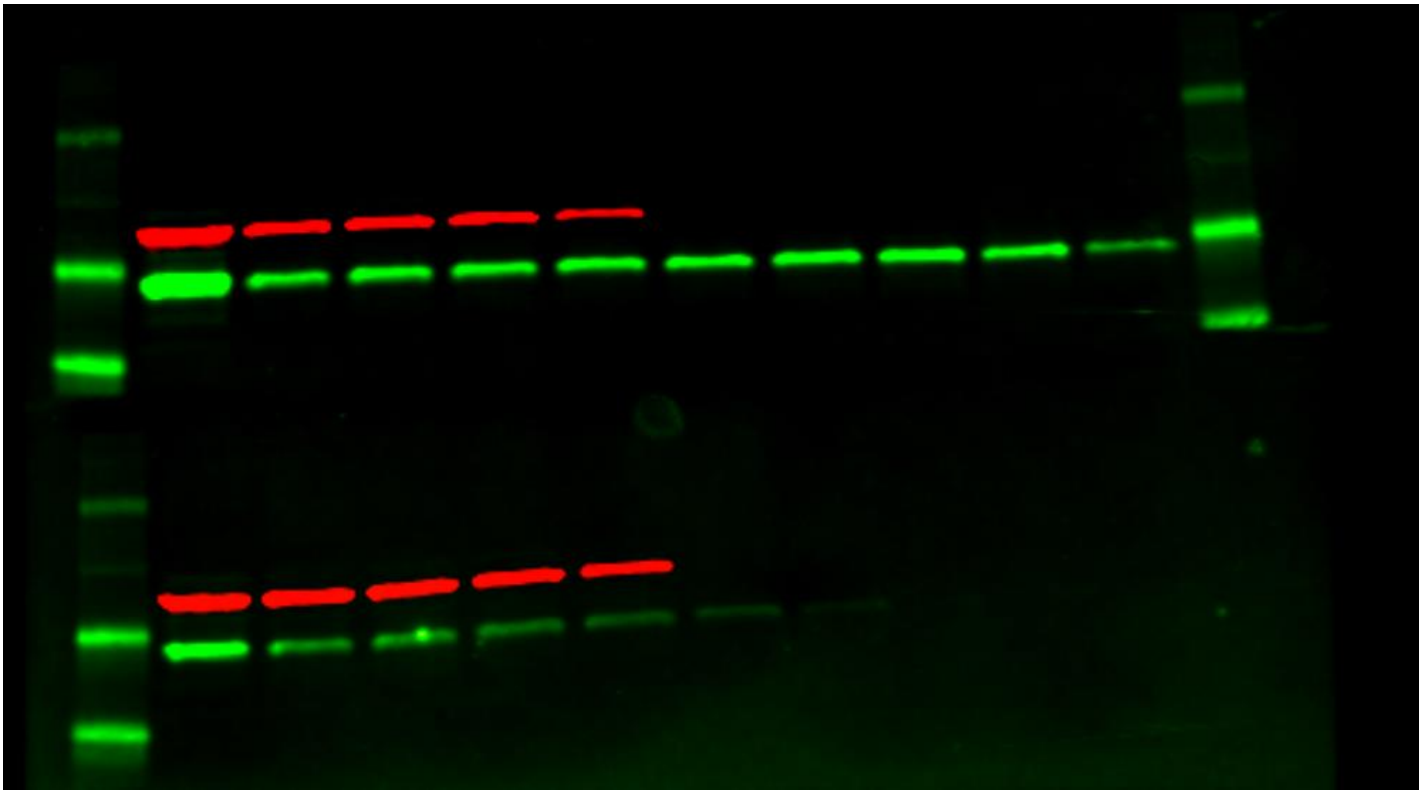

overexposed, ladder fully visible, used to assign MW

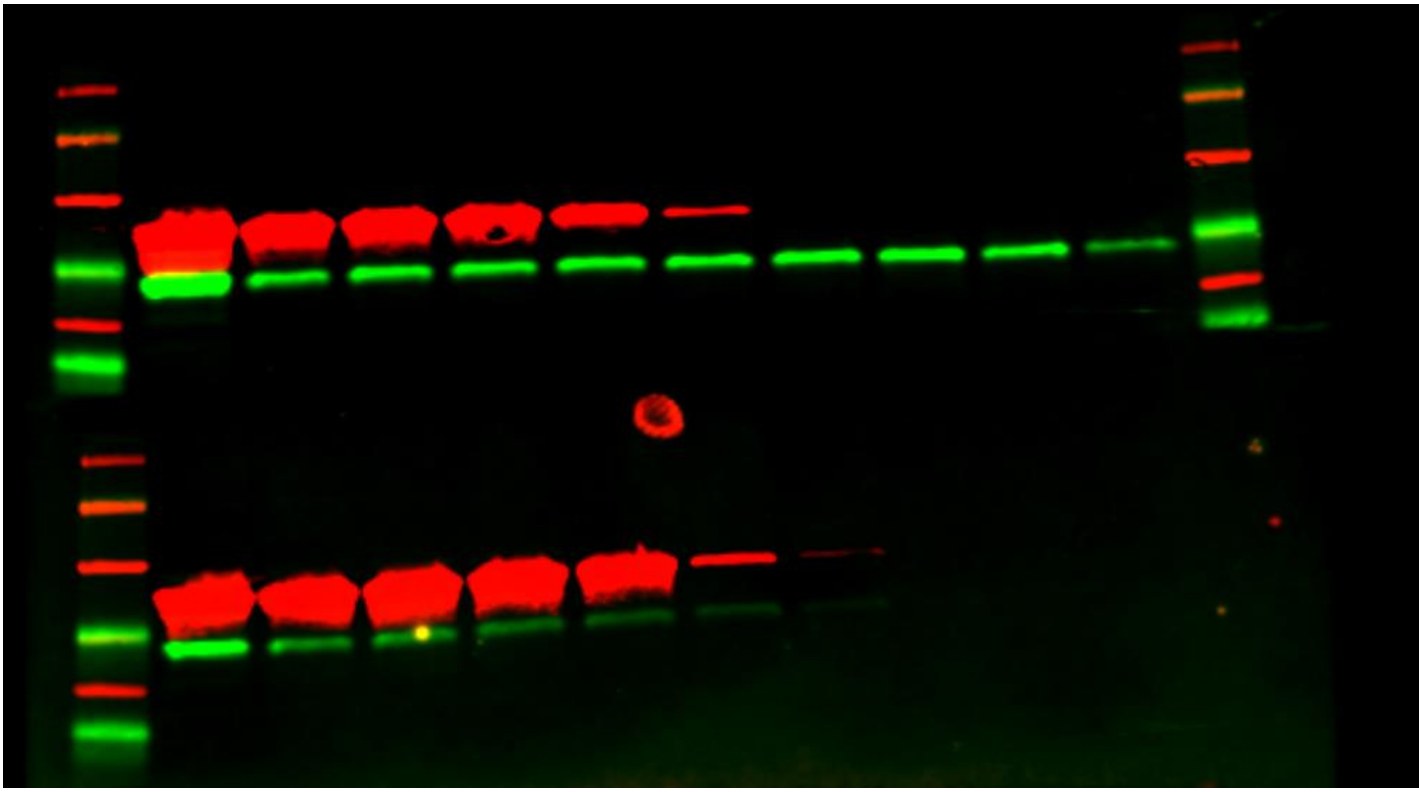

Corresponding to Figure 4B. Green = WDR5.

Upper panel C3TD078

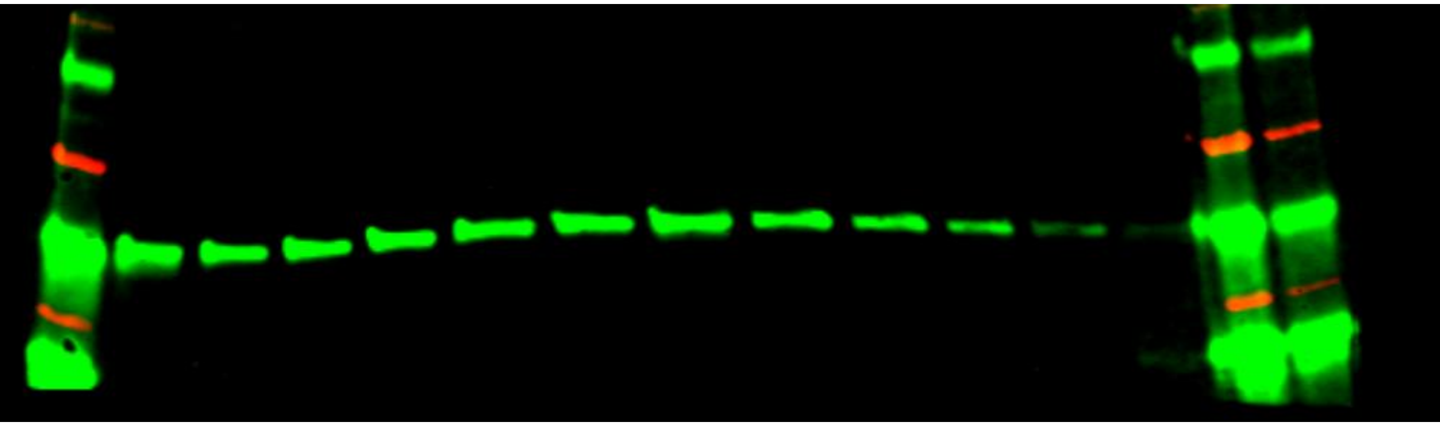

Lower panel C3TD343

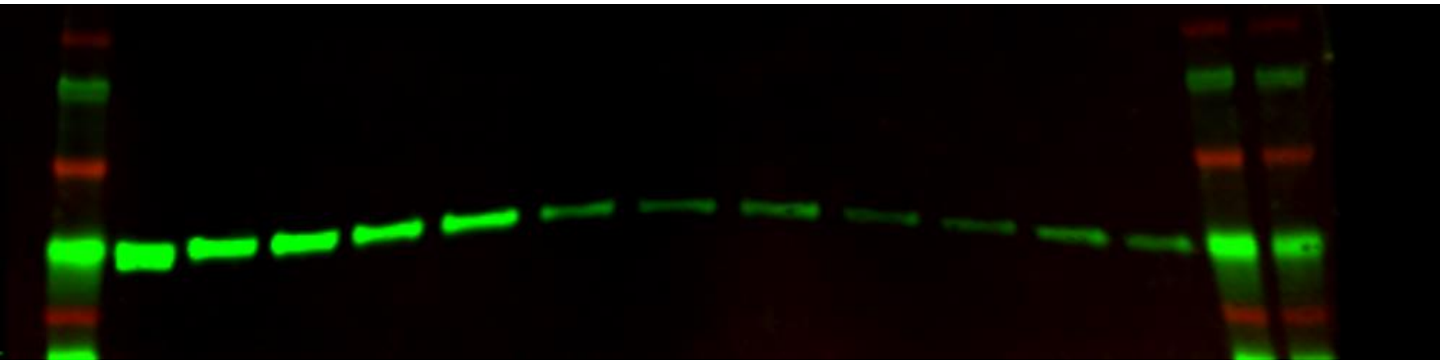

Corresponding to Figure 4D. Green = WDR5, red = ACTN

Upper panel C3TD078, lower panel DMSO

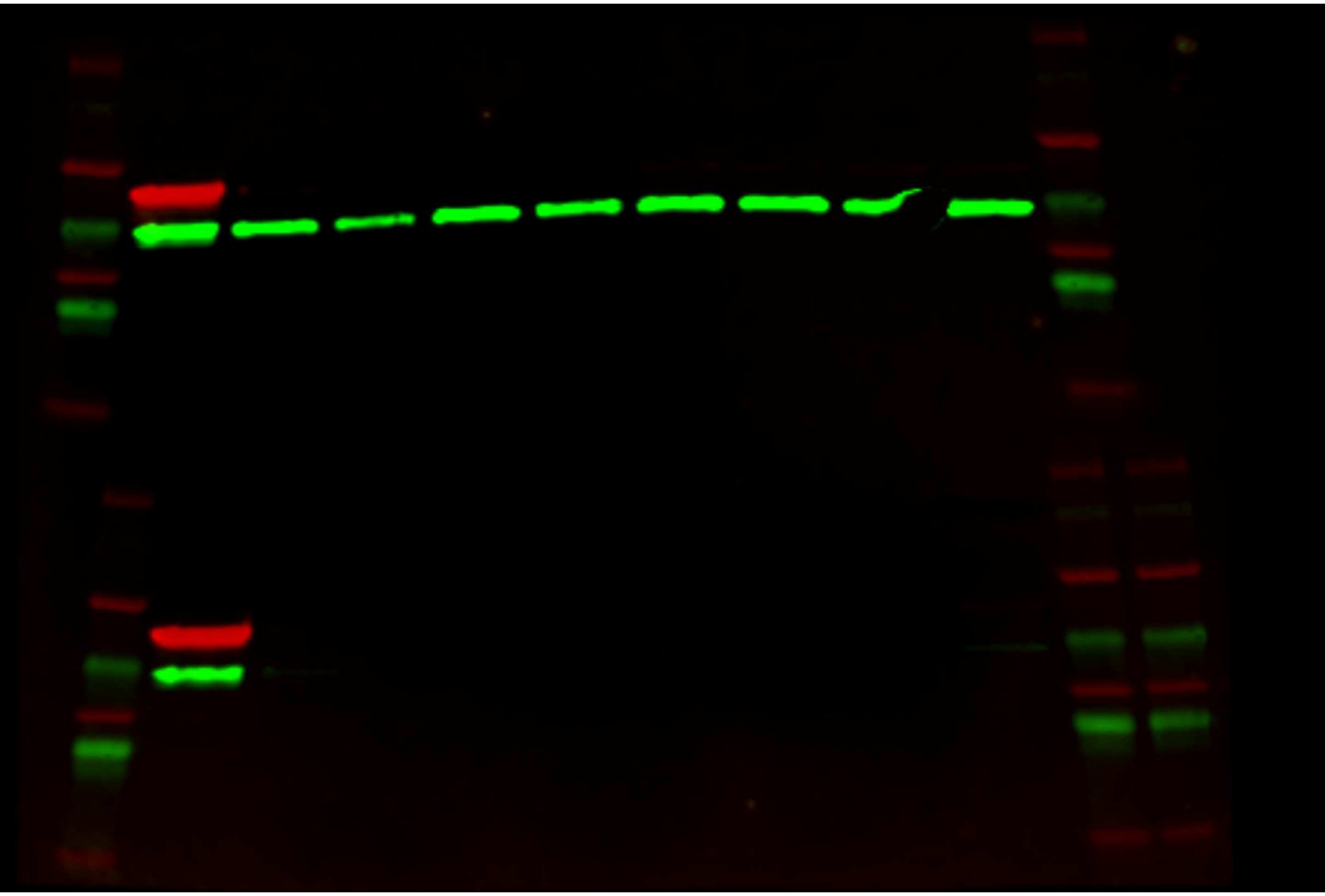

Supplement: Unedited blot and gel images [file jciinsight-11-198298-s301.pdf]
